# Sustainable Applications of Endophytic Bacteria and Their Physiological/Biochemical Roles on Medicinal and Herbal Plants: Review

**DOI:** 10.3390/microorganisms11020453

**Published:** 2023-02-10

**Authors:** Phumudzo Patrick Tshikhudo, Khayalethu Ntushelo, Fhatuwani Nixwell Mudau

**Affiliations:** 1 Department of Agriculture, Land Reform and Rural Development, Directorate Plant Health, Division Pest Risk Analysis, Arcadia, Pretoria 0001, South Africa; 2 Department of Agriculture and Animal Health, College of Agriculture and Environmental Sciences, University of South Africa, Private Bag X6, Florida 1710, South Africa; 3 School of Agricultural, Earth and Environmental Sciences, University of KwaZulu-Natal, Private Bag X01, Scottsville, Pietermaritzburg 3209, South Africa

**Keywords:** antimicrobials, secondary metabolites, plant growth promotion, phosphate solubilization, nitrogen fixation, phytoremediation

## Abstract

Bacterial endophytes reside within the tissues of living plant species without causing any harm or disease to their hosts. These endophytes can be isolated, identified, characterized, and used as biofertilizers. Moreover, bacterial endophytes increase the plants’ resistance against diseases, pests, and parasites, and are a promising source of pharmaceutically important bioactives. For instance, the production of antibiotics, auxins, biosurfactants, cytokinin’s, ethylene, enzymes, gibberellins, nitric oxide organic acids, osmolytes, and siderophores is accredited to the existence of various bacterial strains. Thus, this manuscript intends to review the sustainable applications of endophytic bacteria to promote the growth, development, and chemical integrity of medicinal and herbal plants, as well as their role in plant physiology. The study of the importance of bacterial endophytes in the suppression of diseases in medicinal and herbal plants is crucial and a promising area of future investigation.

## 1. Introduction

It is well known that users of endophytic bacteria employ comparable strategies to enhance plants growth. In addition, endophytic bacteria are more successful than rhizobacteria at reducing the negative impacts of environmental stressors on plants. They enter the plant tissue primarily through the roots and other natural openings in the plant. After entering the plant, bacterial endophytes may spread throughout the tissues of the host plant [1]. Medicinal and herbal plants have secondary metabolites which function as important drugs, flavor, fragrances, agrochemicals, dye, pigments, pesticides, and may play a key role in the adaptation of plants to their environment [2].

Bacterial endophytes of the genera *Bacillus*, *Pantoea*, *Pseudomonas*, *Stenotrophomonas*, and *Serratia* can produce phytohormones, such as auxin and gibberellin, as well as protease and hydrogen cyanide, and play a role in siderophore production, phosphate solubilization, and atmospheric nitrogen fixation [3]. Among other endophytes, *Bacillus*, *Pseudomonas*, *Agrobacterium*, and *Flavobacterium* species solubilize the inorganic phosphate compounds by phosphatases. Bacterial species of the genera *Alcaligenes*, *Arthrobacter*, *Azotobacter*, *Bradyrhizobium*, *Chromobacterium*, *Enterobacter*, *Escherichia*, *Micrococcus*, *Streptomyces*, *Serratia*, and *Thiobacillus* secrete organic acids to solubilize the insoluble phosphorus. *Pseudomonas* spp. and *Bacillus* spp. are known endophytes that are involved in siderophores production [4]. For example, secondary metabolites produced by endophytic bacterium *Bacillus pumilus* have a significant inhibitory effect against fungal species, such as *Pythium aphanidermatum*, *Rhizoctonia solani*, and *Sclerotium rolfsii* [5]. The leaf and stem of heart-leaved moonseed (*Tinospora cordifolia* (Thunb.) Miers) contain *Bacillus*, *Aneurinibacillus*, and *Pseudomonas* species [6]. Traditional medicine has long utilized *T. cordifolia* to treat several conditions including fever, jaundice, chronic diarrhea, cancer, dysentery, bone fractures, pain, asthma, skin diseases, deadly bug bites, and eye issues.

Nitrogen-fixing bacterial strains are linked with certain leguminous and non-leguminous species [7]. Species such as *Gluconacetobacter diazotrophicus* and *Azorhizobium* caulinodans can fix nitrogen [8]. *Pseudomonas putida*, *Azospirillum brasilense*, and *Enterobacter cloacae* enable plants to grow and survive under higher levels of polyaromatic hydrocarbons (PAHs) through phytoremediation [9]. *Arthrobacter* sp. and *Bacillus* sp. bacterial endophytes produce secondary metabolites for plants to adapt to abiotic stress [10]. Several studies have shown that endophytic bacteria play a beneficial role in plant growth. It is not yet clear which bacterial strains contribute more to the growth and development of medicinal plants. It is well-known that the density of endophytic bacteria within the plant tissues is less than that of found in the rooting zone [11]. Plant-growth-promoting (PGP) bacteria that fix nitrogen may be used as biofertilizers to improve plant growth [12]. Many of these bacteria also produce phytohormones [13]—for example, as the bacterial strain SCCPVE07—that improve the growth of coriander (*Coriandrum sativum* L.) grown under salinity stress [14]. The stem and leaves of coriander have antimicrobial and antibacterial qualities. Inoculated *C. sativum* showed higher levels of calcium, carbon, iron, and potassium contents. In this case, the levels of cinnamic acid, 4-methoxy-cinnamic acid hexoside, 5-O-caffeoylquinic acid, K-3-O-rutinoside, Q-3-O-rutinoside, Q-3-O-glucoside, and Q-3-O-glucuronide were significantly increased [15]. In addition, certain plant species are capable of accumulating heavy metals and soil contaminants in their tissues [16].

In this context, we aim to provide a review of the sustainable applications of endophytic bacteria and their physiological and biochemical processes on m medicinal and herbal plants to lay a foundation for future studies intended for the isolation, identification, and characterization of endophytic bacteria from the internal tissues of medicinal and herbal plants. Understanding these functions might lead to the development of new techniques in agricultural and biotechnological environments, specifically for the future cultivation and commercialization of indigenous medicinal and herbal plants.

## 2. Potentialities of Endophytic Bacteria

**Figure 1:**
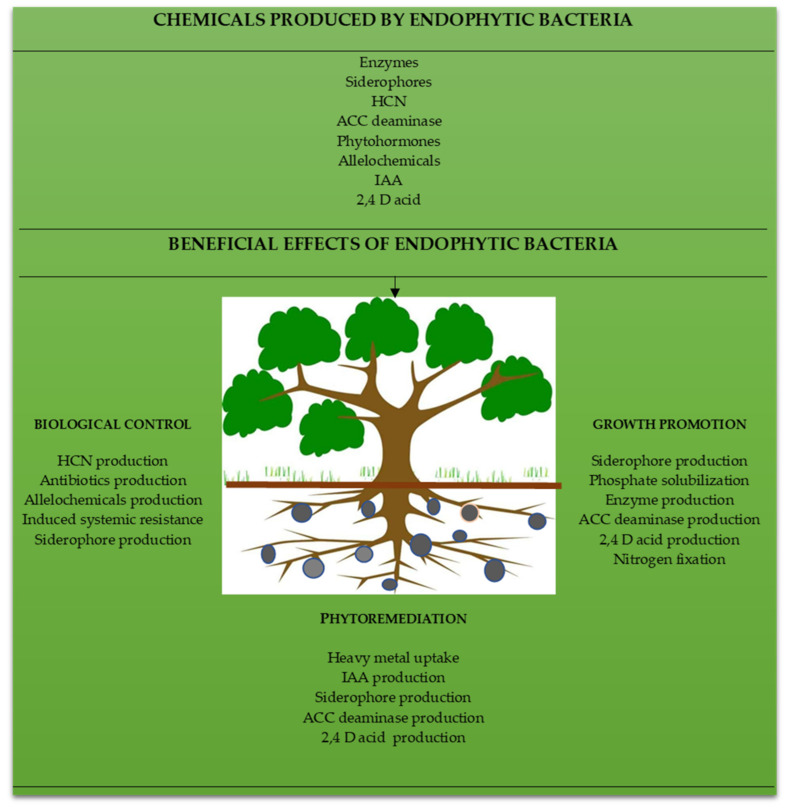
Roles of endophytic bacteria on plant growth, protection, and phytoremediation.

In this regard, this research thoroughly examines several bacterial endophytes and their potentialities on herbal plant species without inflicting any harm or disease to their hosts, drawing on a variety of sources in the literature. These endophytes can be isolated, recognized, and characterized in addition to being employed as biofertilizers to improve plant development and change the chemical makeup of the plant. Additionally, bacterial endophytes boost plants’ resilience to diseases, pests, and parasites and are a promising source of bioactives with the potential for use in pharmaceuticals (Figure 1). For instance, the existence of distinct bacterial strains is credited with the synthesis of antibiotics, auxins, biosurfactants, cytokinins, ethylene, enzymes, gibberellins, nitric oxide organic acids, osmolytes, and siderophores. In order to enhance the growth, development, and chemical integrity of medicinal and herbal plants, as well as their function in plant physiology, this manuscript reviews sustainable applications of endophytic bacteria. It is critical to examine the role of bacterial endophytes in the control of illnesses in medicinal and herbal plants—a promising avenue of future research. 

### 2.1. Growth-Promoting through Nitrogen Fixation, Phosphate Solubilization and Anti-Pathogenic Capabilities

Endophytic bacteria have been increasingly associated with the ability to suppress a broad pool of plants diseases, with this ability being closely associated with the vegetative growth and chemical composition of the plant [17]. Different alkaloids contribute to plant defense through endophytes action, acting as growth-promoting compounds or having a key role in plants’ resistance to environmental stress. Amines and amides, very common metabolites from endophytes, present toxic activities against insects. Steroids, terpenoids, and diterpenes are also generated by endophytes [18] and are responsible of several activities such as plant-growth promotion and yield, suppression of pathogen-growth and colonization, contaminants remotion, phosphate solubilization, and nitrogen plant-assimilation [19]. These properties may be helpful in organic tea plantation [20,21].

*Pseudomonas* and *Bacillus* spp. were studied for their role against blister blight disease caused by *Exobasidium vexans* in tea plants [22]. *Pseudomonas fluorescens*, applied at 7-day intervals, reduced the disease incidence for two seasons to the same level as a fungicide and increased tea yield. Chemical analysis of the plant showed that there was induction of phenolics compounds and defense enzymes, such as peroxidase, polyphenol oxidase, phenylalanine ammonia lyase, chitinase, and β-1,3-glucanase. *Methylobacterium radiotolerans* MAMP 4754 was isolated and identified from the seeds of the therapeutic plant *Combretum erythrophyllum* in order to explore the endophyte’s antibacterial and antioxidant properties [23]. Basil (*Ocimum sanctum* L.) plants have antimicrobial, immunomodulatory, antistress, anti-inflammatory, antipyretic, anti-asthmatic, hypoglycaemic, hypotensive, and analgesic properties [24,25].

Endophytes living within a healthy plant are a good source of antimicrobial agents, enzymes, and secondary metabolites [24]. For example, endophytic bacterial strains isolated from *O. sanctum* leaf tissues are antagonistic to pathogenic fungi, such as *Alternaria solani*, *Fusarium solani*, *R. solani*, *S. rolfsii*, and *Colletotrichum lindemuthianum* [26], and are therefore capable of promoting the *O. sanctum* growth. Interestingly, *O. sactum* treatment with these bacterial endophytes increased the content of plant essential oil, with this demonstrated influence of bacterial endophytes on *O. sactum* growth and oil content [26]. Bacterial endophytes inhibited six out of ten test microorganisms [24]. Among these, *Salmonella typhi*, which is a typhoid-causing bacterium, was significantly inhibited by 3 bacterial isolates (BTS 2, GTL 3, BTS 4), while 2 bacterial isolates (BTS 2, GTL 3) significantly inhibited *Micrococcus luteus*, an opportunistic pathogen. Medicinal species, such as seepweed (*Suaeda nudiflora* Thwaites), white-flowered black mangrove *(Lumnitzera racemose* Willd.), beach moonflower (*Ipomoea tuba* (Schltdl.) Colla), and mangrove (*Avicennia alba* Blume) have different levels of antimicrobial activities against *M. luteus*, *Pseudomonas aeruginosa*, *Staphylococcus aureus*, *Klebsella pneumonia*, and *Bacillus subtilis* [27].

In China, *Glycyrrhiza* spp. are regarded as the most important herbs to treat blood disorders, cancer, and hepatitis, and these plant species are thought to improve the immune system while reducing chemotherapy-related side effects [28]. *Glycyrrhiza* spp. have antimicrobial activity [29]. Licorice (*Glycyrrhiza glabra* L.) is a perennial shrub that contains glabridin which is specifically derived from the plant’s roots [30]. *G. glabra* is used to manage ulcer and respiratory problems [31]. Licorice (*Glycyrrhiza uralensis* Fisch. ex DC) is a leguminous herb that is native to Asia [32], but it is now grown in many parts of the world [32]. The roots of *G. uralensis* produce a variety of terpenoids and flavonoids. Bacterial strains were isolated from the root nodules of *G. uralensis* and *G. glabra* to identify and classify them, as well as to determine their level of stress tolerance [30,32]. Based on 113 physiological and biochemical characteristics, the isolates were clustered into three groups. One bacterial isolate, which belongs to the genus *Mesorhizobium*, has a high tolerance to NaCl, pH, and high temperatures.

Nodulation tests demonstrated that this isolate also formed nitrogen-fixing nodules on other plants, namely sophora (*Sophora viciifolia* Hance), bird’s-foot trefoil (*Lotus corniculatus* L.), white clove (*Trifolium repens* L.), melilot (*Melilotus suaveolens* Ledeb.), and bitter licorice (*Sophora alopecuroides* L.). *S. alopecuroides* has been used as a traditional Chinese medicine to treat fever and diarrhea, among other diseases, as well as to inhibit cancer cell growth [33,34]. 16S rRNA and recA gene analyses revealed the presence of *Agrobacterium tumefaciens*, *Mesorhizobium alhagi*, *Mesorhizobium gobiense*, *Mesorhizobium amorphae*, *Phyllobacterium trifolii*, *Rhizobium giardinii*, *Rhizobium indigoferae*, *Sinorhizobium fredii*, and *Sinorhizobium meliloti* from the root nodules of *S. alopecuroides* in different regions of China’s Loess Plateau [35].

Ginger (*Zingiber officinale* Roscoe) is an important medicinal plant, producing aromatic rhizomes which are valuable both as spice and herbal medicine [36]. Ginger has been used as a traditional medicine in China and India for more than 25 centuries [37]. However, ginger wilt provoked by *Ralstonia solanacearum* is a severe disease which threatens the productivity of ginger in China. Ginger has anti-inflammatory, anti-emetic, and chemo-protective properties [38]. Bacterial strains were isolated from the plant surface as well as the stem, leaf, and root tissues of *Z. officinale* for their antimicrobial activity against R. solanacearum using amplified ribosomal DNA restriction analysis (ARDRA) fingerprint analysis [36]. It was also found that the isolated bacterial strains had the ability to produce certain enzymes and metabolites which exert a significant reduction in disease occurrence when the antagonists were applied.

Rooibos tea (*Aspalathus linearis* (Burm.f.) R.Dahlgren) is the source of commercialized rooibos tea, which is endemic in the mountains of the Western Cape province in South Africa [39]. *Burkholderia tuberum* was originally isolated from *Aspalathus* sp. (rooibos tea plant) nodules in South Africa [40]. Sequence analysis of the 16S rRNA showed that endophytic bacterial isolates were beta-rhizobia [39]. Rooibos tea contains no colorants, additives, or preservatives and is free of caffeine. Bush tea (*Athrixia phylicoides* DC.) is indigenous to Southern Africa. *A. phylicoides* is used for cleansing or purifying blood, treating boils, headaches, infected wounds, and cuts [41,42]. Vhavenda people believe bush tea has aphrodisiac properties [43]. Traditionally, Zulu people prefer bush tea decoction extracted from the root as a cough remedy and purgative [44], whereas the Sotho people use bush tea as a soothing wash for sore feet [45,46]. It is known that leaves of bush tea contain 5-hydroxy-6,7,8,3′,4′,5′-hexamethoxy flavon-3-ol [41,47], 3-O-demethyldigicitrin; 5,6,7,8,3′,4′-hexamethoxyflavone and quercetin [48], total polyphenols [41,48,49], tannins [49], and total antioxidants [50,51,52].

*Echinacea* spp. contain polysaccharides, glycoproteins, alkamides, volatile oils, and flavonoids [53]. The echinacea herb is used for the treatment of flu and colds [54]. Endophytic bacteria of the genera *Acinetobacter*, *Bacillus*, *Pseudomonas*, *Wautersia (Ralstonia*), and *Stenotrophomonas* were identified and characterized from the medicinal plant Echinacea spp. by means of 16S rRNA gene analyses and other microbiological tests such as thin-layer chromatography (TLC) and high-performance liquid chromatography (HPLC). It was also discovered that the *Pseudomonas stutzeri* P3 strain produces the plant indole acetic acid [40]. In terms of antibiotic resistance, it was found that most endophytic bacterial isolates were resistant to the antibiotic kanamycin.

*Bacillus*, *Brachybacterium*, *Kocuria*, *Leucobacter*, *Lysinibacillus*, *Mycobacterium*, *Paenibacillus*, *Pseudomonas*, *Providencia*, *Rhizobium*, and *Streptomyces* have been isolated from *Polygonum cuspidatum* Sieb. et Zucc. and identified using 16S rDNA sequence. *P. cuspidatum* produces polydatin, a glycosylated derivative of resveratrol. These endophytic bacteria isolated from *P. cuspidatum* have inhibitory activity against *Aspergillus niger*, *Aspergillus fumigatus*, *B. subtilis*, *Gibberella fujikuroi*, *K. pneumoniae*, and *S. aureus* [55]. From the roots and leaves of Radish (*Raphanus sativus* L.) at Jinju, Korea, *Proteobacteria*, *Bacillus,* and *Bacteroidetes* spp. were isolated using phylogenetic analysis based on 16S rDNA sequences. *B. subtilis* exhibited amylase, cellulase, xylanase, mannase, PGAase, DNase, protease, and esterase and inhibitory action against plant pathogenic fungi [56]. In China, *R. sativus* is used to treat hypertension, chronic tracheitis, and constipation.

Extract of endophytes isolated from creat or green chiretta (*Andrographis paniculata* (Burm. f.) Wall. ex Nees) contains antibacterial activities against human pathogenic bacteria [57]. From internal tissues of the root and stem of *Piper nigrum* L., endophytic bacterial species such as *Pseudomonas* spp., *Serratia* spp., *Bacillus* spp., *Arthrobacter* spp., *Micrococcus* spp., and *Curtobacterium* sp. (1 strain) were isolated and identified based on 16S rDNA sequencing and found to be effective antagonistic endophytes for the biological control of *Phytophthora capsici* [58]. White Tree Peon (*Paeonia ostia* T. Hong and J.X.Zhang) is an important medicinal plant of China, and 56 endophytic bacterial strains were identified by 16S rDNA gene sequence analysis. These bacterial endophytes contain non-ribosomal peptide synthetase and putative polyketide synthase genes responsible for bioactivities [59].

*Pseudomonas aeruginosa* isolated from balloon flower (*Platycodon grandiflorum* (Jacq.) A. DC.) degraded 2,4-Dichlorophenoxyacetic acid which is responsible for antimicrobial activity against pathogenic fungus causing balloon flower root rot [60]. Endophytic bacteria isolated from the leaves of pennywort (*Centella asiatica* (L.) Urb.), *B. subtilis* BCA31, and *P. fluorescens* BCA08 reduced the growth rate and disease incidence of the causal agent of anthracnose *Colletotrichum higginsianum* [61]. The endophyte *B. subtilis* ALB629 isolated from cacao seedlings has antimicrobial properties against fungi *Moniliophthora perniciosa* and *Colletotrichum gossypii. B. subtilis* ALB629 promotes growth of both the aerial and roots of cacao seedlings [62]. 16S rRNA sequence analysis revealed *Bacillus*, *Streptomyces*, *Pseudovibrio*, and *Pseudomonas* species isolated from *Rhizophora stylosa*. These isolates had antimicrobial activity against *E. coli* ATCC 25922, *P. aeruginosa* ATCC 25923, *B. subtilis* ATCC 27212, *S. aureus* ATCC 12,222, and *C. albicans* ATCC 7754 [63].

*Dendrobium* spp. are medicinal plants containing properties with anti-cancer, anti-fatigue, gastric ulcer protective effects, etc. Of all the bacterial endophytes isolated from stems of *Dendrobium* spp. plants, *Bacillus megaterium* exhibited the highest antimicrobial effects [64]. *Acinetobacter guillouiae*, *B. cereus*, *Burkholderia tropica*, *Novosphingobium* sp., *Pseudomonas moraviensis*, *Pseudomonas* sp., *Rahnella aquatilis*, and *Raoultella ornithinolytica*, were isolated from the Cape coast lily (*Crinum macowanii* Baker) bulbs and showed potential for possible drug lead against common pathogenic bacteria [65]. Traditional uses of *C. macowanii* include treating boils, diarrhea, fever, inflammation, respiratory issues, skin rashes, tuberculosis, wounds, and urinary tract issues. Bacterial isolates FjR1 and FjF2 from the Indian coffee plum (*Flacourtia jangomas* (Lour.) Raeusch.) displayed potential antimicrobial activity against pathogenic bacteria [66]. The longevity spinach (*Gynura procumbens* DC.) is a medicinal plant species for treatment of cancer, constipation, fever, kidney diseases, rheumatism, rashes, headache, and viral skin diseases [67,68]. The leaves of *G. procumbens* contain anti-herpes simplex virus, antihyperglycemic, anti-inflammatory, antihyperlipidemic, anti-allergy agent, and antihypertensive properties [68]. Apart from its antihypertensive, glucose-lowering, and anti-inflammatory properties, it is also a source of proteins and peroxidase [69]. *G. procumbens* leaves contain essential oil, flavonoids, miraculin, polyphenols, peroxidase, thaumatin-like proteins, terpenoids, and unsaturated sterols. The screening of endophytic bacteria for the plant growth regulators such as cytokinin were needed as means to explore if these endophytic bacteria can be applied in agriculture [67]. *Acenitobacter calcoaceticus*, *Paenibacillus polymaxa*, and *Psuedomonas resinovorans* were isolated from *G. procumbens* leaves collected in Malaysia [68]. It was also found that broth extracts from *P. resinovorans* and *P. polymaxa* contain cytokinin-like compounds [68].

Acetone extract from kauri booti (*Ajuga bracteosa* Wall ex Benth.) has antibacterial activity against *E. coli* [70]. This plant contains phytochemicals which have anti-inflammatory, astringent, diuretic, and depurative properties, and can be used to treat agues, menorrhea, bronchitis, diarrhea, fever, gout, jaundice, pneumonia, palsy, and rheumatism [71]. The leaves, bark, stem, and roots of *A. bracteosa* have medicinal properties, can be used as an astringent against hypoglycaemic and gastrointestinal disorders, and have anthelmintic, diuretic, antifungal, anti-inflammatory, and antimycobacterial compounds [71]. Bacterial species were isolated and screened for PGP and biotechnological potential associated with *A. bracteosa*, with most isolates belonged to *Proteobacteria* and *Pseudomonas* [70,71]. These isolates exhibited PGP through production of siderophores and indole acetic acid. They are also capable of phosphate solubilization through production of hydrolytic enzymes such as amylase, cellulose, chitinase, lipase, pectinase, phosphatase, and protease [71].

The biological compounds produced by endophytic bacteria play a pivotal role in the protection of medicinal and herbal plants against pests and pathogens (Table 1). Living plants are a good source for bacterial endophytes which can be isolated for more efficient production of antimicrobial compounds with pharmacological importance. In turn, medicinal plants could also benefit in terms of growth promotion with less application of inputs such as fertilizers, fungicides, insecticides, or herbicides.

### 2.2. Growth-Promoting Bacteria on Medicinal and Herbal Plants

Sixty-one bacteria were identified and comparatively characterized from the root nodules of *Medicago*, *Melilotus Onobrychis*, *Oxytropis*, and *Vicia* species grown in the Loess Plateau and Qinghai-Tibet Plateau [100]. *Vicia sativa* has been used as a traditional medicine to treat asthma, bronchitis, skin infections, and urinary diseases, and it also has anti-poison, antiseptic, aphrodisiac, antipyretic, and antirheumatic properties [101]. *Oxytropis* spp. contain flavonoids, alkaloids, and saponins which have medicinal properties [102]. Apigenin, caffeic acid, gallic acid, pyrogallol, salicylic acid, naringenin, quercetin, myricetin, and daidzein are major secondary metabolites in the extract of the alfalfa (*Medicago sativa* L.) leaves [103]. *Melilotus* spp. are native to the Mediterranean area [104]; their herbs have aromatic, emollient, and carminative properties [105]. *Onobrychis* genus is an important legume with over 150 species [106]. Among the bacterial isolates are *Rhizobium leguminosarum*, *S. meliloti*, and *S. fredii*; the rest belong to *Mesorhizobium*, *Phyllobacterium*, and *Stenotrophomonas.* Species of *R. leguminosarum* was isolated from *Oxytropis* spp. and medick or burclover (*Medicago archiducis-nicolai* Širj.), while *S. fredii* was isolated from the black medick (*Medicago lupulina* L.) grown in the Qinghai-Tibet Plateau. These strains were also found to be resistant to high alkalinity and a high concentration of NaCl [100]. *V. sativa* contains significant activity against pathogenic bacterial species such as *Bacillus atrophaeus*, *E. coli*, *S. aureus*, and *Staphylococcus epidermidis* [101]. Medicago spp. are used to treat eczema, anemia, constipation, body odor, infections, burns, athlete’s foot, cancer, arthritis, intestinal ulcers, gastritis, liver diseases, bleeding gums, and high blood pressure.

The hyacinth bean *(Lablab purpureus* (L.)) serves as a herbal medicine in China for the treatment of internal heat fever. The leaves and fruits of *L. purpureus* contain sterols and fatty acids such as palmitic, palmitoleic, linoleic, and linolenic acids [104,107,108]. Pyridine alkaloids, trigonelline, and sterols have been isolated from tissue cultures of seeds, stems, and leaves. To study the diverse rhizobia associated with this plant, *Bradyrhizobium*, *Rhizobium*, *Ensifer*, and *Mesorhizobium* species were isolated from southern China [104].

Based on 16S rDNA analysis, the bacteria isolated belonged to *Aeromonas*, *Bacteroides*. *Cytophaga*, *Flexibacter*, *Ilyobacter*, *Pelomonas*, *Proteobacteria*, *Pseudomonas*, *Rhodoferax*, *Rhizobium*, *Sulfurospirillum*, and *Uliginosibacterium*. These endophytic bacteria have the capacities of fixing nitrogen and removal of contaminants from the water body through phytoremediation by degrading catechol, cyanide, methane, methanol, methylated amines, oxochlorate, urea, and 2,4-Dichlorophenoxyacetic acid [109]. Although this area of study will not form part of our investigation, we may opportunistically assess the possibility of environmental remediation by bacteria when we survey natural populations of *A. phylicoides*.

*Caragana* spp. are leguminous plants containing more than 80 species worldwide [110]. The roots, flowers, shoots, barks, and seeds are useful plant parts applied as herbal medicine [111] for the treatment of cancer of gynecological problems [112]. Species of the genus *Caragana* are resistant to extreme temperatures and have nitrogen-fixing abilities [110]. *Agrobacterium*, *Mesorhizobium*, *Rhizobium*, *Bradyrhizobium*, and *Phyllobacterium* species are associated with *Caragana* species grown in China [110].

Bacterial species belonging to Actinobacteria, Bacteroidetes, Firmicutes, and Proteobacteria phyla are salinity tolerant nitrogen-fixing endophytic bacteria isolated from roots of the halophyte *Suaeda* sp. obtained in Iran and are capable of growth–promotion the halophyte sea-blite (*Suaeda maritima* (L.) Dumort.) [113]. Nitrogen-fixing bacteria of *Brachybacterium saurashtrense* sp. nov., *Zhihengliuella* sp., *Brevibacterium casei*, *Haererehalobacter* sp., *Halomonas* sp., *Vibrio* sp., *Cronobacter sakazakii*, *Pseudomonas* spp., *Rhizobium radiobacter*, and *Mesorhizobium* sp. were isolated and identified from roots of halophytic pickleweed (*Salicornia brachiatas* Miq.) using analysis of 16S rRNA genes. It has been revealed that isolated endophytic bacteria from *S. brachiatas* is capable of growth promotion based on production of indole acetic acid production, phosphate solubilization, and 1- aminocyclopropane-1-carboxylic acid deaminase [114].

Using 16 S rDNA, it was discovered that the root nodules of *Mimosa patida* contains *Burkholderia* spp. that secretes phytohormone, aminocyclopropane-1-carboxylic acid deaminase, and solubilizes phosphate and has antimicrobial activity against phytopathogens [115]. *Azotobacter beijerinckii*, *Azotobacter chroococcum*, *Azospirillum lipoferum*, and *Azotobacter vinelandii* isolated from the roots of medicinal aloe (*Aloe vera* (L.) Burm.f.) and devil’s trumpet (*Datura metel* L.) fix nitrogen. These isolates also secrete glucose, sucrose, lactose, maltose, rhamnose, xylose, and mannitol. The production of indole acetic acid by *A. beijerinckii*, *A. chroococcum,* and *A. vinelandii* stimulated the growth of *A. vera* and *D. metel* [116].

The root nodules of sulla (*Hedysarum carnosum* L.), clover bord (*Hedysarum spinosissimum* L.), and *Hedysarum pallidum* Desf., sampled from various localities in Algeria, were isolated based on the ARDRA, Random Amplified Polymorphic DNA (RAPD) fingerprinting, and 16S rDNA have, *E. cloacae*, *Enterobacter kobei*, *Escherichia vulneris*, *Leclercia adecarboxylata*, *Pantoea agglomerans* and *Pseudomonas* sp. [117]. *Rhizobium tropici* isolated had nitrogen-fixing symbioses with *Medicago ruthenica* [118]. Both the isolation and characterization of *Rhizobium*, *Sinorhizobium*, *Mesorhizobium*, and *Bradyrhizobium* species from *Acacia*, *Anthyllis*, *Argyrolobium*, *Astragalus*, *Calycotome*, *Coronilla*, *Ebenus*, *Genista*, *Hedysarum*, *Hippocrepis*, *Lathyrus*, *Lotus*, *Medicago*, and *Ononis* species from southern Sudan were based on comparative 16S ARDRA using seven enzymes, total cell protein sodium dodecyl sulphate–polyacrylamide gel electrophoresis (SDS-PAGE) analysis, and 16S rDNA sequencing [119].

*Rhizobium huautlense*, a nitrogen-fixing rhizobial symbionts of bigpod sesbania (*Sesbania herbacea* (Mill.) McVaugh), sampled at Sierra de Huautla, Mexico, was isolated and identified using PCR-RFLP analysis. The electrophoretic alloenzyme types (Ets) were also identified [120]. *R. indigoferae* sp. nov. and *Sinorhizobium kummerowiae* sp. nov from root nodules of *Indigofera* spp. and *Kummerowia* spp.; *R. loessense* sp. nov from the root nodules of *Astragalus* and *Lespedeza* spp; and *R. sullae* sp. nov were described as the nitrogen-fixing bacterial symbionts [121,122,123]. The multi-locus enzyme electrophoresis (MLEE) and 16S rRNA gene sequence analysis revealed two nitrogen fixing bacterial species of *Sinorhizobium arboris* sp. nov. and *Sinorhizobium kostiense* sp. nov. from the root nodules of *Acacia rutico* and *Prosopis chilensis* sampled in Sudan and Kenya [124].

*Sinorhizobium morelense* sp. nov. isolated from root nodules of jumbay (*Leucaena leucocephala* (Lam.) de Wit) has been found to be resistant to carbenicillin, kanamycin, and erythromycin [125]. This medicinal plant is frequently used to treat diabetes. It is also used to treat stomach ailments, assist abortion, and promote contraction. The revealed *Allorhizobium undicola* sp. nov. solated from the water mimosa or sensitive Neptunia (*Neptunia natans* (Girard) Kuntze) based on the 16S rRNA gene sequencing has nitrogen-fixing ability [126]. This medicinal plant has pharmacological qualities that include antifungal, antiemetic, astringent, anthelmintic, antidysentery, diuretic, anti-inflammatory, antioxidant, hypercholesterolemia, antipyretic, and antiemetic properties.

From nodules of False Indigo (*Amorpha fruticose* L.), *M. amorphae* sp. nov was characterized based on the RFLP of PCR-amplified 16S rRNA genes, MLEE, DNA–DNA hybridization, 16S rRNA gene sequencing, electrophoretic plasmid profiles, cross-nodulation, and a phenotypic study [127]. *A. fruticose* is used to treat dermatitis, carbuncles, and burns in traditional Chinese medicine. DNA–DNA hybridizations were conducted to identify *Mesorhizobium* spp. from the sample from the white carob tree (*Prosopis alba* Griseb.) root nodules grown in Argentina [128]. *R. tropici* were found to be root modulating bacteria of *N. natans* from India [129]. Prosopis plants have a variety of bioactive qualities, including antioxidant, anti-inflammatory, anti-cancer, and anti-diabetic properties.

A new species name for a root-forming nodule bacterial isolate, *Devosia neptuniae* sp. nov., which was previously classified as *A. undicola*, was suggested to neptunium-modulating rhizobia isolated from India. This bacterial species can form a bonafide dinitrogen-fixing root-nodule symbiosis with other legume plants [130].

Root-nodule isolates of *Bradyrhizobium*, *Mesorhizobium*, *Rhizobium*, and *Sinorhizobium* spp. from *Lespedeza* spp., collected from China and the USA, were isolated and characterized using SDS-PAGE analysis of whole-cell proteins, DNA–DNA hybridization, and 16S rRNA gene sequence analysis [131]. *Ralstonia taiwanensis* sp. nov. was isolated from *Mimosa* spp. and is the capable of root nodule formation and nitrogen fixation [132].

The endophytic bacterial isolates from *H. carnosum*, *H. spinosissimum* subsp. *capitatum*, and *H. pallidum* grown in Algeria were identified as *P. agglomerans*, *E. kobei*, *E. cloacae*, *L. adecarboxylata*, *E. vulneris*, and *Pseudomonas* sp. by means of ARDRA using the enzyme Cfo I, RAPD fingerprinting and sequencing of 16S rDNA. This was the first report confirming that Gammaproteobacteria is associated with *H. carnosum*, *H. spinosissimum* subsp. *Capitatum*, and *H. pallidu* [117]. The diversity of endophytic bacteria from the root of Angelica sinensis in Angelica in the Gansu province revealed that certain endophytic bacterial strains may have come from the rooting zone [133].

Among the endophytic bacteria isolated from the red clover (*Trifolium pratense* L.), *P. agglomerans* (59.6%) was mostly detected in foliage tissues, *Agrobacterium rhizogenes* A in the tap root (49.2%), and *R. leguminosarum* BV *phaseoli* and *R. loti* B in the nodules (27.2% each) [134]. In addition, *T. pratense* has been used medicinally to treat a number of illnesses, such as eczema and psoriasis, cancer, whooping cough, respiratory issues, and skin inflammations. *B. megaterium*, *Bordetella avium*, *Curtobacterium luteum*, and *R. leguminosarum* BV *trifolii* promoted the growth of *T. pratense*. Nodulation of red clover seedlings became evident because of co-inoculation of *R. leguminosarum* BV *trifolii* with *Bacillus insolitus*, *B. brevis*, or *A. rhizogenes* A [134]. Crop rotations of *T. pratense* and potato (*Solanum tuberosum* L.) have specific associations with bacterial endophytes. Of all the growth-promoting bacterial strains isolated from these plants, 63% enhanced shoot height, 66% enhanced shoot wet weight, and 55% enhanced root wet weight [135]. It has been claimed that potatoes offer a multitude of medicinal properties, including antioxidant, anticancer, antiallergy, antibacterial, anti-inflammatory, anti-obesity, and anti-ulcer action. A total of 200 bacterial isolates from the berseem clover (*Trifolium alexandrinum* L.) possess plant growth promoting traits. Production of indole acetic acid by the endophytic bacteria stimulated the plant development of rice plant [136]. Because of their expectorant, analgesic, and antibacterial qualities, *Trifolium* spp. are also used to treat rheumatic pains. Co-inoculation of *T. repens* with *Rhizobium* strains CHB1120 and CHB1121, *Bacillus aryabhattai* strain Sb, and *A. vinelandii* strain G31 promotes the nitrogen fixation and nutrient uptake of white clover in a P-deficient soil [137].

Nitrogen-fixing bacteria can form endophytic colonies in various medicinal and herbal plants. These beneficial bacteria fix nitrogen from the atmosphere to enhance the length and biomass of medicinal and herbal plants. Nitrogen-fixing endophytic bacteria may be useful for the sustainable production of medicinal and herbal plants, specifically in saline-based environments. Nitrogen-fixing bacteria are currently being introduced as biofertilizers.

Native phosphate-solubilizing endophytic bacteria, including those from rhizospheres, improve the growth of herbal and medicinal plants. Various leaf structural and chemical characteristics were assessed as possible predictors of the size of the phyllosphere bacterial population associated with plant species such as strawberry tree (*Arbutus unedo* L. 1753), lesser calamint (*Calamintha nepeta* (L.) Kuntze), rockrose (*Cistus × incanus* L.), lavender (*Lavandula stoechas* L.), lemon balm (*Melissa officinalis* L.), common myrtle (*Myrtus communis* L.), lentisk or mastic (*Pistacia lentiscus* L.), and kermes oak (*Quercus coccifera* L.) [57]. *A. unedo* contain polyphenols, which are reported to manage cancer risk, coronary heart disease, and other degenerative diseases [58]. *Quercus* spp. have been used as traditional medicinal plants and are antiseptic and hemostatic for the treatment of diarrhea and hemorrhoids [59]. Certain species of the genus *Quercus* contain antimicrobial, anti-inflammatory, gastroprotective, antioxidant, cytotoxic, and antitumor properties [59]. The extract of the different parts of *P. lentiscus* has antiatherogenic, anti-inflammatory, antioxidant, antimicrobial, hypotensive, anticancer, anti-arthritis, antigout, anti-asthmatic, and anthelmintic activities [60]. *M. communis* is used for various ailments such as diarrhea, dysentery, gastric ulcers, hemorrhages, leucorrhea, rheumatism, sinuses, and vomiting [61]. Lavender oils are derived from the true lavender (*Lavandula angustifolia* Mill.), Spanish lavender (*Lavandula stoechas* L.), broadleaved lavender (*Lavandula latifolia* Medik.) and lavender (*Lavandula × intermedia* ‘Grosso’) [62], which have antimicrobial, anti-inflammatory, hypnotic, and anxiolytic properties [63]. *C. × incanus* contains phenolic substances together with an associated strong antioxidant activity [64]. *C. nepeta is* aromatic, diaphoretic, expectorant, febrifuge, and stomachic [65]. *M. officinalis* possesses antioxidants such as caffeic and rosmarinic acids [66]. *M. officinalis* also contains antibacterial and antiviral properties. The role of the P-solubilizing bacterium *Enterobacter ludwigii* in the growth promotion and P content of barley (*Hordeum vulgare* L.) was investigated [138].

The study revealed beneficial effects on the dry weight, P assimilation, and barley yield in *E. ludwigii*-inoculated plants [138]. *C. nepeta*, *M. communis*, *M. officinalis*, and *L. stoechas* produce essential oils Interestingly, aromatic plants are more colonized than the other species, whereas the non-woody perennials are more highly colonized than the water pepper (*Polygonum hydropiper* (L.) Delabre 1800) is regarded as a P-accumulating herb used for P-phytoextraction, with higher P-accumulating capability in the mining ecotype compared to the non-mining ecotype [139]. In traditional medical systems, water pepper is used as an astringent, sedative, antiseptic, and to treat respiratory problems, edema, and snake bites.

Tea plant (*Camellia sinensis* (L.) Kuntze) is used in one of the most important beverages; the plant contains about 4000 bioactive compounds, of which 1/3 is contributed by polyphenols alone [140]. The phytochemical screening of green tea showed the presence of alkaloids, saponins, tannins, catechin, and polyphenols [56,141], which are quality parameters of tea. A study was conducted to examine the diversity of cultivable P-solubilizing bacteria in *C. sinensis* [142]. Over 900 rhizoplane bacteria were randomly selected and were identified using fatty acid methyl ester (FAME) analysis and isolates belonging to *Bacillus* (34.6%), *Pseudomonas* (8.9%), *Stenotrophomonas* (6.1%), *Paenibacillus* (5.9%), and *Arthrobacter* (4.8%) were identified [2]. The leaf of *C. sinensis* showed antimicrobial properties against *S. aureus*, *Vibrio cholerae*, *C. jejuni*, *S. epidermidis*, *V. mimicus*, *L. monocytogenes*, *E. coli*, *S. typhi*, *P. aeruginosa*, *H. pylori*, and *P. acnes*, among others [143,144,145,146,147,148,149,150,151,152]. *Proteobacteria* and *Sphingobacteria* spp. possess plant growth promoting attributes such as indole acetic acid, aminocyclopropane-1-carboxylic acid deaminase production, and P and K-solubilization [153].

Aloe *(Aloe barbadensis* Mill.) is an important medicinal plant with applications in pharmaceutical, food, and cosmetic industries and is used for flavoring liquid formulations [154]. *A. barbadensis* contains phenolic compounds such as aloin-A (barbaloin), aloesin, soaloeresin D, and aloeresin E used in the treatment of tumors, diabetes, ulcers, and cancer [155]. *Burkholderia gladioli*, *Enterobacter hormaechei*, *Pseudomonas synxantha*, and *S. marcescens* isolated from *A. barbadensis* are capable of solubilizing phosphate into liquid phase [154]. From *Brassica juncea* (L). Czern and Coss var. Pusa Bold (DIR-50), a Ni-tolerant *B. subtilis* strain SJ-101 was isolated and identified. It was found that the strain SJ-101 produced indole acetic acid and solubilized inorganic phosphate [55]. Endophytic bacteria isolate from *P. hydropiper* contains plant growth promotion traits including indole acetic acid, siderophores, and phosphate-solubilization among others [156]. Endophytic bacterial strains isolated from the tissues of livening thyme (*Thymus vulgaris* L.) exhibited some plant growth-promoting activities such as auxin synthesis, diazotrophic, P-solubilization, siderophore production, and production of lytic enzymes (i.e., chitinase, cellulase, protease, and lipase) under in vitro conditions [157]. *T. vulgaris* has been used for treating chest congestion and promoting salivation since ancient times. The fresh leaves are also consumed to soothe sore throats.

Endophytic bacteria are capable of solubilizing inorganic phosphate in a solid medium, thereby increasing its availability to living medicinal and herbal plants (Table 2). Phytochemicals released by endophytic bacteria promote the sustainable production of medicinal and herbal plants resulting from improved fertility of the soil, and hence increase medicinal and herbal plant production.

### 2.3. Endophytic Bacterial Species Responsible for Phytomediation on Medicinal/Herbal Plants

Due to a variety of industrial activities as well as natural processes, the accumulation of heavy metals in soil has rapidly grown. Endophytic bacteria can be isolated from leaves, stems, and roots of plants grown in contaminated soil. Many endophytes are capable of degrading organic contaminants and heavy metals, and can therefore be used for phytoremediation on contaminated soils [175]. The mobility and bioavailability of heavy metals in the soil influence phytoextraction and phytostabilization [16,155]. Plant tolerance to the contamination is crucial for successful phytoremediation [176]. Decontamination may be accelerated with appropriate microorganisms’ inoculation that is able to break down pollutants and compete with indigenous microorganisms.

Yellowtop *(Alyssum murale* Waldst. and amp; Kit.) is regarded as a metal hyperaccumulator plant and is able to solubilize Ni. *A. murale* has been used in combination with other medicinal plants for gynecological disorders. It has also been found that *Microbacterium oxydans* AY509223 increased Ni uptake of *A. murale* [177]. NBRI K28 *Enterobacter* sp. also has aminocyclopropane-1-carboxylic acid deaminase activity and increased the growth of the brown mustard (*Brassica juncea* (L.) Czern.) plants. NBRI K28 *Enterobacter* sp. enhanced phytoextraction of metals of Ni, Zn, and Cr accumulated by *B. juncea* [178,179]. *P. putida* strain PS9, isolated from the turnip (*Brassica campestris* L.), solubilized phosphate and produced significant amount of salicylic acid, 2,3-dihydroxy benzoic acid, and indole acetic acid [180]. Antioxidant, antibacterial, and anticancer properties are present in *Brassica* spp.

A considerable number of bacterial strains have been isolated from heavy metal-polluted soil in Nanjing, China, which promoted plant growth and cadmium uptake in rape [*Brassica napus* L.) [181]. Some of these bacterial isolates had the potential to solubilize cadmium carbonate in solution culture. It has been also confirmed that these cadmium-resistant isolates exhibit the presence of indole acetic acid. These bacterial isolates colonized and developed in *B. napus* after root inoculation [181]. *P. fluorescens* G10 and *Microbacterium* sp. G16 were isolated and identified by means of 16S rDNA gene sequence analysis from the roots of *B. napus* grown in Pb-contaminated soils. There was significant increase in root elongation of inoculated *B. napus* seedlings. Endophytic bacterium JN6 isolated from roots of the drooping knotweed (*Polygonum pubescens* Blume) was identified as *Rahnella* sp. This showed very high Cd, Pb, and Zn tolerance and effectively solubilized CdCO3, PbCO3, and Zn3(PO4)2 in culture solution [10]. *Microbacterium* sp. G16 produced indole acetic acid, siderophores, and 1-amino cyclopropane-1-carboxylate deaminase [181]. It has been demonstrated that *P. pubescens* works well as a traditional Chinese medicine. The *Proteobacteria*, *Actinobacteria*, and *Bacteroidetes* Chloropid isolated from the goat willow (*Salix caprea* L.) were resistant to Zn/Cd as they produced aminocyclopropane-1-carboxylic acid deaminase, indole acetic acid, and siderophores [182]. The leaves *S. caprea* are made into a decoction which is used to cure fevers. The isolated *Bradyrhizobium* sp. (vigna) RM8 from nodules of *V. radiata* sampled from nickel and zinc in India promoted the growth of the host plant [183]. This was evidently shown by the increase in nodule numbers, leghaemoglobin, seed yield, grain protein, root N, and shoots.

Using HPLC analysis, *Enterobacter* sp. was isolated from the long-stamen onion (*Allium macrostemon* Bunge) plants grown in polycyclic aromatic hydrocarbon-contaminated soils [184]. This bacterial species also promoted the growth of wheat and maize and removed pyrene from pyrene-amended soil in pot experiments. *Enterobacter* sp. produced indole acetic acid, siderophore, and solubilize inorganic phosphate. *A. macrostemon* has historically been used to alleviate thoracic pain, stenocardia, heart asthma, and diarrhea. *Bacillus* sp., isolated from the roots of venboksal (*Alnus firma* Siebold and Zucc.) using 16S rRNA sequence analysis, demonstrated the capacity to produce siderophores and indole acetic acid. There was increased root elongation of inoculated *B. napus* seedlings [185]. The isolates facilitated the capability of reducing heavy metal phytotoxicity and increasing Pb accumulation in *A. firma*. *Alnus* spp. are well recognized for their traditional medical uses, which include treating conditions including cancer, hepatitis, uterine cancer, rheumatism, and dysentery, as well as causing stomachaches, diarrhea, and fever. The bacterial population associated with *T. caerulescens* subsp. *calaminare* sampled had Zn and Cd capabilities due to the increased availability of the metals in soils near the roots [186]. Based on 16S rRNA sequence analysis, *Methylobacterium* spp., *Rhodococcus* spp., and *Okibacterium* spp. were isolated from the pennycress (*Thlaspi goesingense* Halácsy) accumulated Ni in ultramafic soils. These isolates produced 1-amino cyclopropane-1-carboxylic acid deaminase and siderophore [187]. Four groups of heavy metal-resistant bacterial such as *Actinobacteria*, *Proteobacteria*, *Bacteroidetes*, and *Firmicutes* were isolated from the roots, stems, and leaves of black nightshade (*Solanum nigrum* L.) [188]. These isolates were re-inoculated into *S. nigrum* under Cd stress which resulted in Cd phytotoxicity decrease. *S. nigrum* has historically been used to treat bacterial infections, coughs, and indigestion. An endophytic bacteria *Serratia* sp. RSC-14 isolated from the roots of *S. nigrum* displayed phosphate solubilization and produced indole acetic acid [189].

Endophytic bacteria associated with the roots, stems, and leaves of *Alyssum bertolonii* Desv. sampled from central Italy influenced plant growth [190]. These endophytic bacteria also shown potential for nickel-hyperaccumulation There was significant increase in biomass and metal accumulation. *Cupriavidus taiwanensis* TJ208 isolated from the bashful (*Mimosa pudica* L.) removed Pb, Cu, and Cd from polluted soils [191]. Since ancient times, *M. pudica* has been administered topically to heal wounds as well as urogenital disorders, piles, dysentery, and sinuses. *Bacillus thuringiensis* GDB-1 enhanced growth of *A. firma*, through production of aminocyclopropane-1-carboxylic acid deaminase activity, indole acetic acid, and siderophores; as well as P solubilization. *B. thuringiensis* GDB-1 also accumulated As, Cu, Pb, Ni, and Zn in seedlings of *A. firma* [192]. Zn-tolerant bacterial strains such as *B. subtilis*, *B. cereus*, *Flavobacterium* sp., and *P. aeruginosa*, isolated from the Chinese violet cress (*Orychophragmus violaceus* (L.)), significantly increased the shoot biomass and Zn accumulation in *O. violaceus* [3]. On the other hand, *B. subtilis*, *B. cereus*, *B. megaterium*, and *P. aeruginosa* isolated from *O. violaceus* significantly enhanced growth plant and Cd accumulation [193]. *O. violaceus* oil can be used to make a variety of cosmetic products for the care of the skin, hair, and lips, as well as to make external preparations for the treatment of burns. The diversity of endophytic bacteria associated with the root, stem, and leaf of *Poplus* sp. enhanced phytoremediation on localities contaminated with BTEX compounds [194].

From *S. nigrum* grown in metal-polluted soil, *Acinetobacter* sp. LSE06, *Enterobacter aerogenes* LRE17, *Enterobacter* sp. LSE04, and *Serratia nematodiphila* LRE07 isolates possesses aminocyclopropane-1-carboxylic acid deaminase, indole acetic acid, siderophores, and P solubilizing activity. *Acinetobacter* sp., *E. aerogenes*, *Enterobacter* sp., and *S. nematodiphila* significantly increased Cd extraction from the soils [195]. From a pot experiment *Pseudomonas* sp. Lk9 isolated from *S. nigrum* produces biosurfactants, siderophores, and organic acids [196]. *Serratia* sp. isolated from *S. nigrum* was resistant to the toxic effects of heavy metals through the production of indole acetic acid, siderophore, and solubilized mineral phosphate [197]. Species of *Microbacterium*, *Arthrobacter*, *Agreia*, *Bacillus*, *Sthenotrophomonas*, *Kocuria*, and *Variovorax* isolated from the roots of *Noccaea caerulescens* displayed significant increases in shoot biomass, root length, and root-to-shoot Ni translocation [198]. *B. pumilus* E2S2 isolated from the stonecrop (*Sedum plumbizincicola* X.H.Guo and S.B.Zhou ex L.H.Wu) improved its phytoextraction capacity [199]. Common stonecrop is used by people for a variety of things, including coughing, high blood pressure, and wound healing. Endophytic bacterial species isolated from the Chinese brake (*Pteris vitata* L.) and the spider brake (*Pteris multifida* Poir.) improved arsenic tolerance and speciation in plants [200]. *Pteris* spp.’s bioactive potential includes some cytotoxic, anticancer, antiproliferative, neuroprotective, wound-healing, antibacterial, antiviral, hepatoprotective, leishmanicidal, trypanocidal, antinociceptive, anti-inflammatory, immunomodulatory, and chemopreventive properties.

Root-colonizing beneficial bacteria can improve plant growth through resistance to biotic and abiotic stresses. Drought is an environmental condition affecting the productivity of medicinal plants globally. PGP rhizobacteria produce secondary metabolites that could relief drought stress in plants [201]. *Pseudomonas pseudoalcaligenes* and *B. pumilus* have a significant ability to withstand adverse effects caused by saline stress [202].

In vitro inoculation of grapevine (*Vitis vinifera* L.) explants with *Burkholderia phytofirmans* increased grapevine growth and physiological activity at a low temperature [203]. Root zone bacterial species such as *Arthrobacter*, *Azotobacter*, *Azospirillum*, *Bacillus*, *Enterobacter*, *Pseudomonas*, *Serratia*, and *Streptomyces* produce compounds that benefit living plants through abiotic stress relief [204]. *Streptomyces coelicolor*, *S. geysiriensis*, and *S. olivaceus* are drought tolerant plants grown in arid and drought-affected regions [205]. It has been established that proanthocyanidin-rich *V. vinifera* seed extract protects against a wide range of illnesses, including infections, cancer, diabetes, hypertension, peptic ulcers, and cardiovascular disease. These endophytic actinobacteria produce phytohormones, contain PGP traits, and have the water-stress tolerance potential to promote growth plants grown in stressed environments. *P. fluorescens* and *B. subtilis* isolated from *V. radiata* produce water-stress-related proteins and enzymes [206]. *B. saurashtrense* and *Pseudomonas* sp. isolated from *Salicornia brachiate* significantly increased the host’s growth under salt stress conditions [55]. *Methylobacterium* sp. (strain BJ001) isolated from *Populus deltoides* × *nigra* DN34 degraded 2,4,6-trinitrotoluene (TNT), hexahydro-1,3,5-trinitro-1,3,5-triazene (RDX), and octahydro-1,3,5,7-tetranitro-1,3,5-tetrazocine (HMX) [56,158]. Rheumatism, arthritis, lower back pains, urinary complaints, digestive and liver problems, debility, anorexia, fevers, and menstrual cramp discomfort are all traditionally treated with *Populus* spp. [207]. *Bacillus*, *Pseudomonas*, *Klebsiella*, *Serratia*, *Arthrobacter*, *Streptomyces*, *Isoptericola*, and *Microbacterium* species isolated from the leaves, stems and roots of sea lavender (*Limonium sinense* (Girard) Kuntze) produced Aminocyclopropane-1-carboxylic acid deaminase and indole acetic acid. They are also capable of N2-fixation and phosphate-solubilization [57]. *Limonium* species have antibacterial, antioxidant, free radical-scavenging, and antiviral properties.

*A. linearis* and *Cyclopia* spp. are South African indigenous herbal leguminous species grown in acidic soils. Among the rhizobial isolates from root nodules of *A. 24spalathus* and *Cyclopia* spp., bacterial species of genera *Rhizobium*, *Burkholderia*, *Mesorhizobium*, and *Bradyrhizobium* produce bioactive compounds that affect the growth of leguminous plants [58].

A heavy-metal-resistant strain of *Bacillus edaphicus* NBT was evaluated on *B. juncea* for its plant growth promotion. *B. edaphicus* NBT produced indole acetic acid, siderophores, and aminocyclopropane-1-carboxylic acid deaminase. There was also an increase in Pb uptake by *B. juncea* inoculated with *B. edaphicus* NBT [208]. In pot experiments, 16S rDNA sequencing identified *E. aerogenes* and *R. aquatilis* isolated from *B. juncea*. These isolates stimulated the growth of *B. juncea* exposed to environments contaminated with Ni and Cr [209]. These bacteria also produced siderophores, aminocyclopropane-1-carboxylic acid deaminase, indole acetic acid, and phosphate solubilization [209]. *B. pumilus* (STR2) and *Exiguobacterium oxidotolerans* (STR36) promoted the growth of the water hyssop (*Bacopa monnieri* (L.) Pennell) grown in salt-stressed soils, enhanced proline levels and decreased lipid peroxidation [76]. Traditional medicine from *B. monnieri* may improve cognitive performance, cure ADHD symptoms, and lessen stress and anxiety.

The jute mallow or nalta jute (*Corchorus olitorius* L.) inoculated with *P. extremorientalis* TSAU6, indole acetic acid, and gibberellic acid exhibited a significant increase in root length, shoot length, and fresh weight, indicating that plant growth regulators such as auxins and gibberellins play an important role in plant salinity tolerance [59]. *C. olitorius* is a leafy vegetable that is frequently used in soup recipes and traditional medicine to treat tumors, chronic cystitis, and fever. *B. megaterium* MTCC446 isolated from gale of the wind (*Phyllanthus amarus* Schumach. and Thonn.) promoted a higher vigor index, germination (%), plant biomass, P content, plant phenolic content, radical scavenging, and antioxidant activity [60]. *P. amarus* is frequently used in African traditional medicine to treat a variety of illnesses, including kidney stones, dysentery, jaundice, diarrhea, and urogenital problems.

Under the pot experiment, *Achromobacter xylosoxidans* isolated from the bright eyes (*Catharanthus roseus* (L.) G. Don) exposed to saline soils in Tamilnadu, India, produced aminocyclopropane-1-carboxylic acid deaminase [210]. *C. roseus* is a significant medicinal plant that is found around the world. It contains a variety of phytochemicals that have biological effects (antioxidant, antibacterial, antifungal, and anticancer). Under drought conditions, the ringed lavender (*Lavandula dentata* L.) isolates of *B. thuringiensis* increased plant growth and nutrition. *B. thuringiensis* produced indole acetic acid and aminocyclopropane-1-carboxylic acid deaminase and solubilized P, demonstrating its capacity to enhance plant growth under stress conditions [102]. In traditional medicine, *L. dentata* has been used to cure rheumatism, headaches, colds, and the flu.

Environmental contamination became a major challenge in recent decades due to rapid industrialization in developed countries. Mining activities, wastewater discharge, volcanic eruptions, and rock weathering affected the growth and productivity of soil. Endophytic bacterial species could be a potential mechanism in phytoremediation strategies for the management of environmental contaminants, while plants benefit through growth promotion (Table 3).

## 3. Conclusions

The sustainable production of medicinal and herbal plants has been one of the major challenges facing agriculture in recent years, with the ongoing over-utilization of chemicals to meet the population demands. To solve these problems, an environmentally friendly way forward focusing on the minimal usage of agrochemicals is require. Endophytic bacterial isolates could be a potential source of phytochemical compounds to enhance plant production. In addition, endophytic bacteria enable the plant species to resist abiotic stress conditions. Endophytic bacterial living in plant tissues produce plant growth-promoting compounds such as phytohormones; enzymes such as Aminocyclopropane-1-carboxylic acid deaminase, which reduce the levels of ethylene; organic acids aiding in P solubilization; and siderophores, cellulases, and chitinases inhibiting the phytopathogens growth. Sustainable agriculture provides a platform to medicinal and herbal plant producers from which to apply new agricultural techniques and biotechnologies to enhance plant growth by using bacterial isolates as biofertilizers. The modes of actions of several endophytic bacterial species have been well documented; they are recognised as plant growth promotion agents based on other biochemical and physiological attributes such as biological nitrogen fixation, phosphate solubilization, siderophore production, and the synthesis of PGP substances. In addition to improving plant nutrient availability and providing protection against varied abiotic and biotic stresses, endophytic bacteria play a pivotal role in enhancing medicinal and herbal plant productivity while simultaneously ensuring the sustainable maintenance of soil health.

## Figures and Tables

**Table 1:** Endophytic bacteria with antimicrobial activity and associated secondary metabolites from medicinal and herbal plant species.

Isolated Bacterial Species	Biochemical/Physiological Activity	Host Medicinal Plant Species	Host Plant Part/Region	References
*Pseudomonas* sp., *Bacillus* sp., *Enterobacter* sp., *Pantoea* sp., *Chryseobacterium* sp., *Sphingobacterium* sp., *Aeromonas* sp., *Providencia* sp., *Cedecea* sp., *Klebsiella* sp., *Cronobacter* sp., *Macrococcus* sp., *Shigella* sp.	1,1-diphenyl-2-picrylhydrazyl, ascorbic acid production	*Aloe vera*	Stem, leaf, root	[72]
*Streptomyces* sp., *Micromonospora* sp., *Microbiospora* sp., *Nocardia* sp.	Coronamycin, hydroxamate type siderophores production	*Azadirachta indica*	Stem, leaf, root	[73]
*Bacillus* sp., *Bacillus megaterium*, *Bacillus pumilus*, *Bacillus licheniformis*, *Micrococcus luteus*, *Paenibacillus* sp., *Pseudomonas* sp., *Acinetobacter calcoaceticus*	Amylase, esterase, lipase, protease, pectinase, xylanase, and cellulose production	*Plectranthus tenuiflorus*	Stem, leaf, root	[74]
21 endophytic bacteria	Amines, amides, acids, quinines, indole derivatives, steroids, azoles, alcohols, and hydrocarbons production	*Cleodendrum myricoides*, *Lannea flavus*, *Dichrostachys cinerea*, *Gomphocarpus fruticosus*, *Balanites aegyptica*, *Jasminium floribundum*, *Hibiscus fuscus*	Root	[75]
*Bacillus polyfermenticus*, *Bacillus subtilis*, *Bacillus licheniformis*, *Bacillus pumilus*	Cellulase production, Xylanase production, annanase production Pectinase, Amylase, Protease, Lipase, Esterase, DNase, Chitinase production	*Codonopsis lanceolata*	Root	[76]
*Bacillus amyloliquefaciens*	diethyl ether, ethyl acetate production	*Memecylon edule*, *Tinospora cordifolia*, *Phyllodium pulchellum*, *Dipterocarpus tuberculatus*	Whole plant	[77]
*Bacillus* spp.	NM	*Rumex pulcher*	Leaf, stem	[78]
*Bacillus amyloliquefaciens*	diethyl ether; chloroform and ethyl acetate production	*Tinospora cordifolia*, *Phyllodium pulchellum*, *Dipterocarpus tuberculatus*		[77]
*Bacillus* spp.	NM	*Hypericum scabrum*	Leaf	[78]
*Bacillus licheniformis*, *Bacillus pumilus*	cellulase, xylanase, mannase, pectinase, amylase, protease, lipase, esterase, DNase, linchinase, chitinase production	*Platycodon grandiflorum*	Root	[79]
*Bacillus* sp., *Paenibacillus* sp., *Pseudomonas* sp., *Ralstonia* sp., *Micrococcus* sp., *Alcaligenes* spp.	Catalase, Amylase, Gelatinase, Lipase, Nitrate Reductase, Dextrose, Fructose, Lactose, Maltose and Sucrose production	*Paederia foetida*	Leaf, stem	[80]
*Streptomyces griseus*	p-Aminoacetophenonic acids production	*Kandelia candel*	NM	[81]
*Serratia marcescens*	Oocydin A production	*Rhyncholacis penicillata*	NM	[82]
*Streptomyces* spp.	Munumbicins, Munumbicin D production	*Kennedia nigriscans*	NM	[83]
*Paenibacillus polymyxa*	Fusaricidin A–D production	*Arabidopsis thaliana*, *Canola* spp.	NM	[84]
*Streptomyces* sp.	Coronamycin production	*Monstera* sp.	NM	[85]
*Phyllobacterium* sp.	Fatty acids production	*Epimedium brevicornum*	Stem, root	[86]
*Streptomyces* sp.	Indolosesquiterpenes, xiamycin, indosespene, sespenine production	*Kandelia candel*	Stem	[87]
*Streptomyces* sp.	Xiamycin production	*Bruguiera gymnorrhiza*	Stem	[88]
*Saccharopolyspora flava*, *Pseudonocardia zijingensis*, *Nocardia carnea*, *Streptomyces hainanensis*, *Polymorphospora rubra*, *Janibacter melonis*, *Nocardiopsis dassonvillei*, *Glycomyces sambucus*	NM	*Maytenus austroyunnanensis*	Root	[89]
*Gordonia sputi*, *Gordonia polyisoprenivorans*, *Microbacterium paraoxydans*, *Nocardiopsis dassonvillei*, *Jiangella alkaliphila*, *Tsukamurella tyrosinosolvens*	NM	*Maytenus austroyunnanensis*	Stem	[89]
*Saccharopolyspora gregorii*	NM	*Gloriosa superba*	Stem	[89]
*Amycolatopsis pretoriensis*	NM	*Saccharopolyspora gregorii*	Root	[89]
*Lentzea albida*	NM	*Ficus tikoua*	Root	[89]
*Pseudonocardia alni*	NM	*Callicarpa longifolia*	Leaf	[89]
*Pseudonocardia kongjuensis*	NM	*Lobelia clavata*	Leaf	[89]
*Rhodococcus fascians*	NM	*Cercidiphyllum japonicum*	Leaf	[89]
*Micromonospora narashino*, *Micromonospora peucetia*, *Promicromonospora aerolata*, *Arthrobacter mysorens*, *Actinomadura atramentaria*, *Nonomuraea candida*	NM	*Mycobacterium monacense*	Leaf	[89]
*Dietzia maris*	NM	*Schima* sp.	Stem	[89]
*Dietzia natronolimnaea*	NM	*Cercidiphyllum japonicum*	Root	[89]
*Streptomyces specialis*	NM	*Sedum* sp.	Root	[89]
*Blastococcus aggregatus*, *Dactylosporangium aurantiacum*	NM	*Tripterygium wilfordii*	Root	[89]
*Catellatospora chokoriensis*	NM	*Berberis chingii*	Root	[89]
*Kineosporia aurantiaca*, *Saccharopolyspora flava*	NM	*Tripterygium wilfordii*	Stem	[89]
*Promicromonospora sukumoe*	NM	*Cymbopogon citratus*	Root	[89]
*Oerskovia jenensis*	NM	*Ginkgo* sp.	Leaf	[89]
*Micrococcus flavus*	NM	*Polyspora axillaris*	Root	[89]
*Micrococcus flavus*	NM	*Aquilaria sinensis*	Root	[89]
*Actinocorallia aurantiaca*	NM	*Duranta repens*	Root	[89]
*Actinocorallia herbida*	NM	*Millettia reticulata*	Leaf	[89]
*Herbidospora cretacea*	NM	*Osyris wightiana*	Stem	[89]
*Glycomyces algeriensis*	NM	*Carex baccans*	Root	[89]
*Glycomyces algeriensis*	NM	*Scoparia dulcis*	Root	[89]
*Unidentified bacterial endophytes (gram positive)*	Amines, amides, acids, quinines, indole derivatives, steroids, azoles, alcohols, hydrocarbons production	*Cleodendrum myricoides*, *Lannea flavus*, *Dichrostachys cinerea*, *Gomphocarpus fruticosus*, *Balanites aegyptica*, *Jasminium floribundum*, *Hibiscus fuscus*	Root	[89]
*Lysinibacillus* sp., *Paenibacillus* sp., *Pseudomonas* sp., *Bacillus* sp., *Kocuria* sp., *Streptomyces* sp., *Providencia* sp., *Rhizobium* sp., *Leucobacter* sp., *Brachybacterium* sp., *Mycobacterium* sp.	fengycins, surfactants production	*Polygonum cuspidatum*	Root	[55]
*Enterobacter* sp., *B*. *subtilis*	amylase, cellulase, xylanase, mannase, PGAase, DNase, protease, and esterase production	*Raphanus sativus*	Leaf, root	[55]
*Bacillus* spp.		*Andrographis paniculata*	Leaves	[57]
*Pseudomonas* spp., *Serratia* sp., *Bacillus* sp., *Arthrobacter* sp., *Micrococcus* sp., *Curtobacterium* sp.		*Piper nigrum*	Root, stem	[58]
*Bacillus amyloliquefaciens*	Bacteriocins production	*Bruguiera gymnorrhiza*	Leaf	[90]
*Bacillus thuringiensis*, *B*. *pumilus*		*Suaeda monoica*, *Suaeda maritima*, *Salicornia brachiata*, *Lumnitzera racemosa*, *Sesuvium portulacastrum*, *Rhizophora apiculata*, *Rhizophora mucronata*, *Bruguiera cylindrica*, *Ceriops decandra*, *Avicennia marina*, *Aegiceras corniculatum*	Leaf	[91]
*Paenibacillus* sp.	Cellulases, xylanases, pectinase, fusaricidin, peptide synthetase, lipopeptides, and bacitracin production	*Aloe chinensis*	Root	[92]
*Actinomadura* sp., *Amycolatopsis* sp., *Dactylosporangium* sp., *Kocuria* sp., *Kribbella* sp., *Micrococcus* sp., *Micromonospora* sp., *Nonomuraea* sp., *Promicromonospora* sp., *Pseudonocardia* sp., *Rhodococcus* sp., *Streptomyces* sp., *Streptosporangium* sp.	Antibiotics production	*Artemisia annua*	Leaf, stem, root	[93]
*Streptomyces* sp., *Streptosporangium* sp., *Microbispora* sp., *Streptoverticillium* sp., *Sacchromonospora* sp., *Nocardia* sp.	Munumbacin A-D, Kakadumy, Coronamyncin production	*Azadirachta indica*	Leaf, stem, root	[94]
*Bacillus* sp., *Enterobacter* sp., *Streptomyces* sp.	NM	*Paeonia ostii*	Root	[95]
*Pseudomonas aeruginosa*	2,4-Dichlorophenoxyacetic acid ichlorophenoxyacetic acid, Phenazone production	*Platycodon grandiflorum*	Root	[79]
*Paenibacillus polymyxa*, *Bacillus* sp., *Pseudomonas poae*	Cellulase, xylanase, pectinase production	*Panax ginseng*	Root	[96]
*Bacillus amyloliquefaciens* subsp. *Plantarum*, *Bacillus methylotrophicus*	NM	*Panax notoginseng*	root, stem, Petiole, leaf, seed	[97]
*Bacillus subtilis*, *Pseudomonas fluorescens*	Iturin A, surfactant production	*Centella asiatica*	Leaf	[98]
*Arthrobacter* sp., *Achromobacter* sp., *Bacillus* sp., *Enterobacter* sp., *Erwinia* sp., *Pseudomonas* sp., *Pantoea* sp., *Serratia* sp., *Stenotrophomonas* sp.	Cellulase, protease, β-1,3-glucanase, hydrogen cyanide production	*Hypericum perforatum*	Root, stem, leaf	[99]
*Achromobacter* sp., *Bacillus* sp., *Enterobacter* sp., *Erwinia* sp., *Pseudomonas* sp., *Pantoea* sp.	Cellulase, Protease, β-1,3-glucanase, hydrogen cyanide production	*Ziziphora capitata*	Root, stem, leaf	[99]
*Pseudomonas hibiscicola*, *Macrococcus caseolyticus*, *Enterobacter* *ludwigii*, *Bacillus anthracis*	1,1-diphenyl-2-picrylhydrazyl production	*Aloe vera*	Root, stem, leaf	[72]
*B*. *tequilensis*, *Pseudomonas entomophila*, *Chryseobacterium indologenes*, *B. aerophilus*	Ascorbic acid production	*Aloe vera*	Root, stem, leaf	[72]

NM = not mentioned.

**Table 2:** Growth-promoting bacteria and associated secondary metabolites from medicinal and herbal plant species.

Isolated Bacterial Species	Biochemical/Physiological Activity	Host Medicinal Plant Species	Host Plant Part/Region	References
*Enterobacter* sp. strain 638, *Stenotrophomonas maltophilia R551-3*, *Pseudomonas* putida W619, *Serratia proteamaculans* 568	Root/ biomass development	*Populus* spp.	Root and shoot	[158]
*Bradyrhizobium* sp.	Increased Nitrogen and phosphorus uptake	*Cicer arietinum*	Root	[159]
*Mesorhizobium ciceri*	Increased Nitrogen and phosphorus uptake	*Vigna radiata*	Root	[160]
*Rhizobium* sp.	Indole acetic acid	*Cajanus cajan*	Root	[161]
*Bradyrhizobium* sp.	Increased uptake of P, N and Mg High chlorophyll content	*Vigna radiata*	Root	[162]
*Methylobacterium* sp.	NM	*Crotalaria glaucoides*	Root	[163]
*Azotobacter* sp., *Azospirillum* sp.	indole acetic acid production	*Aloe vera*	Root	[164]
*Pantoea agglomerans*, *Enterobacter kobei*, *Enterobacter cloacae*, *Leclercia adecarboxylata*, *Escherichia vulneris*	NM	*Hedysarum carnosum*, *H*. *spinosissimum* subsp. *capitatum*, *H*. *pallidum*	Root	[117]
*Rhizobium* *leguminosarum*	NM	*Pisum sativum*, *Vicia* sp., *Lathyrus* sp., *Lens* sp., *Trifolium pratense*, *Trifolium* spp., *Phaseolus vulgaris*, *P*. *angustifolius*, *P*. *multiflorus*	NM	[165]
*R*. *elti biovar phaseoli*	NM	*Phaseolus vulgaris*, *Leucaena* sp.	NM	[166]
*R*. *etli biovar mimosae*	NM	*Mimosa affinis*	NM	[167]
*R*. *hainanense*	NM	*Desmodium sinuatum*, *Desmodium gyroides*, *Desmodium triquetrum*, *Desmodium heterophyllum*, *Acacia sinicus*, *Arachis hypogaea*, *Centrosema pubescens*, *Macroptilium lathyroides*, *Stylosanthes guianensis*, *Tephrosia candida*, *Uraria crinita*, *Zornia diphylla*	NM	[168]
*R*. *gallicum biovar gallicum*	NM	*Phaseolus vulgaris*, *Leucaena leucocephala*, *Macroptilium atropurpureum*, *Onobbrychis viciifolia*	NM	[168]
*R*. *gallicum biovar phaseoli*	NM	*Phaseolus vulgaris*	NM	[168]
*R*. *mongolense*	NM	*Medicago ruthenica*, *Phaseolus vulgaris*	NM	[168]
*R*. *galegae biovar orientalis*	NM	*Galega orientalis*	NM	[168]
*R*. *galegae biovar officinalis*	NM	*Galega officinalis*, *Astragalus cruciatus*, *Argyrolobium* *uniflorum*, *Anthyllis henoniana*, *Lotus creticus*, *Medicago* spp.	Root	[168]
*R*. *giardinii biovar giardinii*	NM	*Leucaena leucocephala*, *Macroptilium atropurpureum*	NM	[168]
*R*. *giardinii biovar phaseoli*	NM	*Phaseolus vulgaris*	NM	[168]
*R*. *huautlense*	NM	*Sesbania herbacea*	NM	[168]
*R*. *indigoferae*	NM	*Indigofera amblyantha*, *I*. *carlesii*, *I*. *potanini*	NM	[168]
*R*. *sullae*	NM	*Hedysarum coronarium*	NM	[168]
*R*. *loessense*	NM	*Astragalus* sp., *Lespedeza* sp.	NM	[168]
*R*. *yanglingense*	NM	*Coronilla varia*, *Amphicarpaea trisperma*, *Gueldenstaedtia multiflora*	NM	[168]
*Sinorhizobium meliloti*	NM	*Medigaco* sp., *Melilotus* sp., *Trigonella* sp.	NM	[168]
*S*. *sahelense*	NM	*Sesbania* sp., *Acacia* sp.	NM	[168]
*S*. *terangae*	NM	*Acacia* sp., *Sesbania* sp.	NM	[168]
*S*. *medicae*	NM	*Medicago* sp.	NM	[168]
*S*. *kostiense*	NM	*Acacia* sp., *Prosopis* sp.	NM	[168]
*S*. *morelense*	NM	*Leucaena leucocephala*	NM	[168]
*S*. *americanum*	NM	*Acacia* sp.	NM	[168]
*S*. *arboris*	NM	*Acacia* sp., *Prosopis* sp.	NM	[168]
*S*. *kummerowiae*	NM	*Kummerowia stipulacea*	NM	[168]
*Ensifer adhaerens*	NM	*Sesbania* sp., *Medicago* sp., *Sesbania grandiflora*, *Leucaena leucocephala*, *Pithecellobium dulce*, *Medicagosativa* sp.	NM	[168]
*Allorhizobium undicola*	NM	*Neptunia natans*, *Acacia senegal*, *A*. *seyal*, *A tortilis*, *Lotus arabicus*, *Faidherbia albida*	NM	[168]
*Mesorhizobium loti*	NM	*Lotus corniculatus*, *L*. *tenuis*, *L*. *japonicum*, *L*. *krylovii*, *L*. *filicalius*, *L*. *schoelleri*, *Anthyllis* spp., *Lupinus* spp.	NM	[168]
*M*. *huakuii*	NM	*Astragalus sinicus*, *Acacia* spp.	NM	[168]
*M*. *ciceri*	NM	*Cicer arietinum*	NM	[168]
*M*. *tianshanense*	NM	*Glycyrrhiza pallidiflora*, *G*. *uralensis*, *Sophora alopecuroides*, *Glycine max*, *Swainsonia salsula*, *Halimodendron holodendron*, *Caragana polourensis*	NM	[168]
*M*. *mediterraneum*	NM	*Cicer arietinum*	NM	[168]
*M*. *plurifarium*	NM	*Acacia senegal*, *A*. *seyal*, *A*. *tortilis*, *Leucaena leucocephala*, *L*. *diversifolia*, *Prosopisjuliflora*, *Chamaecrista ensiformis*	NM	[168]
*M*. *amorphae*	NM	*Amorpha fruticosa*	NM	[168]
*M*. *chacoense*	NM	*Prosopis alba*	NM	[168]
*Methylobacterium nodulans*	NM	*Crotalaria podocarpa*, *C*. *Perottetti*, *C*. *glaucoides*.	NM	[168]
*Ochrobactrum* spp.	NM	*A*. *mangium*, *Faidherbia albida*, *Paraserianthes falcataria*	NM	[168]
*Devosia neptuniae*	NM	*Neptunia natans*	NM	[168]
*Azorhizobium caulinodans*	NM	*Sesbania rostrata*	NM	[168]
*A*. *johannense*	NM	*Sesbania virgata*	NM	[168]
*Azorhizobium* sp.	NM	*Sesbania rostrata*	NM	[168]
*Bradyrhizobium japonicum*	NM	*Glycine max*, *Glycine soja*, *Macroptilium atropurpureum*	NM	[168]
*B*. *elkanii*	NM	*Glycine max*, *Vigna* spp., *Macroptilium atropurpureum*	NM	[168]
*B*. *liaoningense*	NM	*Glycine max*, *Glycine soja*	NM	[168]
*B*. *yuanmingense*	NM	*Lespedeza cuneata*	NM	[168]
*B*. *betae*	NM	*Beta vulgaris*	NM	[168]
*B*. *canariense*	NM	*Genisteae and Loteae plants*	NM	[168]
*Bradyrhizobium* sp.	NM	*Lupinus* spp., *Mimosa* spp., *Faidherbia* spp., *Acacia* spp., 27 herb legumes	NM	[168]
*Blastobacter denitrificans*	NM	*Aeschynomene indica*	NM	[168]
*Burkholderia caribensis*	NM	*Mimosa pudica*, *M*. *diplotricha*	NM	[168]
*B*. *tuberum*	NM	*Alysicarpus glumaceus*	NM	[168]
*B*. *phymatum*	NM	*Aspalatus carnosa* *Machaerium lunatum*	NM	[168]
*Ralstonia taiwanensis*	NM	*Mimosa pudica*, *M*. *diplotricha*.	NM	[168]
*Pantoea agglomerans*	NM	*Hedysarum carnosum*, *H*. *spinosissimum* subsp. *capitatum*, *H*. *pallidum*	NM	[168]
*Enterobacter kobei*	NM	*Hedysarum carnosum*, *H*. *spinosissimum* subsp. *capitatum*, *H*. *pallidum*	NM	[168]
*Enterobacter cloacae*	NM	*Hedysarum carnosum*, *H*. *spinosissimum* subsp. *capitatum*, *H*. *pallidum*	NM	[168]
*Leclercia adecarboxylata*	NM	*Hedysarum carnosum*, *H*. *spinosissimum* subsp. *capitatum*, *H*. *pallidum*	NM	[168]
*Escherichia vulneris*	NM	*Hedysarum carnosum*, *H*. *spinosissimum* subsp. *capitatum*, *H*. *pallidum*	NM	[168]
*Pseudomonas* sp.	NM	*Hedysarum carnosum*, *H*. *spinosissimum* subsp. *capitatum*, *H*. *pallidum*	NM	[168]
*Pseudomonas* sp.	Indole acetic acid, hydrogen cyanide, and siderophore production	*Solanum* sp.	Rhizosphere	[169]
*Enterobacter aerogenes* NBRI K24, *Rahnella* *aquatilis* NBRI K3	Aminocyclopropane-1-carboxylic acid deaminase, indole acetic acid, siderophore production	*Brassica juncea*	NM	[170]
*Enterobacter* sp.	Aminocyclopropane-1-carboxylic acid deaminase, indole acetic acid, siderophore production	*Brassica juncea*	NM	[170]
*Pseudomonas aeruginosa*	Aminocyclopropane-1-carboxylic acid deaminase, indole acetic acid, siderophore production	*Solanum nigram*	NM	[171]
*Bacillus thuringiensis* GDB-1	Aminocyclopropane-1-carboxylic acid deaminase, indole acetic acid, Siderophores, Phosphate solubilization	*Alnus firma*	Root, shoot	[172]
*B*. *pumilus* E2S2, *Bacillus* sp. E1S2	indole acetic acid, ACC deaminase, siderophores, phosphate solubilization	*Sedum plumbizincicola*	Root, shoot	[172]
*Pseudomonas tolaasii* ACC23, *P*. *fluorescens* ACC9	ACC deaminase, siderophores indole acetic acidproduction	*B*. *napus*	Root, shoot	[172]
*Pseudomonas veronii*	indole acetic acid, decrease soil pH, supply P, and Fe	*S*. *alfredii*	Root, shoot	[172]
*Rhizobium indigoferae* sp. nov. and *Sinorhizobium kummerowiae* sp.	NM	*Indigofera* spp. and *Kummerowia stipulacea*	Root	[121]
*Achromobacter xylosoxidans* Ax10	Aminocyclopropane-1-carboxylic acid deaminase, indole acetic acidproduction; P solubilization	*B*. *Juncea*	Root, shoot	[172]
*Psychrobacter* sp. SRA1, *Bacillus cereus* SRA10	Aminocyclopropane-1-carboxylic acid deaminase indole acetic acid, P solubilization	*B*. *Juncea*	Root, shoot	[172]
*Pseudomonas* sp. SRI2, *Psychrobacter* sp. SRS8, *Bacillus* sp. SN9	Aminocyclopropane-1-carboxylic acid deaminase, indole acetic acid production; P solubilization,	*B*. *Juncea*	Root, shoot	[172]
*Pseudomonas* sp. SRI2, *Psychrobacter* sp. SRS8, *Bacillus* sp. SN9	Aminocyclopropane-1-carboxylic acid deaminase indole acetic acid production; P solubilization	*Brassica oxyrrhina*	Root, shoot	[172]
*Bacillus subtilis*, *B*. *cereus*, *B*. *megaterium*, *Pseudomonas aeruginosa*	Improve plant growth and root elongation	*Orychophragmus* *violaceus*	Root, shoot	[172]
*Pantoea agglomerans* Jp3-3, *Pseudomonas thivervalensis* Y1-3-9	Indole acetic acid, siderophores, Aminocyclopropane-1-carboxylic acid deaminase production; phosphate solubilization	*B*. *napus*	Root, shoot	[172]
*Enterobacter* sp. JYX7, *Klebsiella* sp. JYX10	Indole acetic acid, siderophores, Aminocyclopropane-1-carboxylic acid deaminase production, phosphate solubilization	*Polygonum pubescens*	Root, shoot	[172]
*Rahnella* sp.	Indole acetic acid, siderophores, Aminocyclopropane-1-carboxylic acid deaminase production; phosphate solubilization	*Amaranthus hypochondriacus*, *A*. *mangostanus*, *Solanum nigrum*	Root, shoot	[172]
*Kluyvera ascorbata* SUD165	Increased biomass; Aminocyclopropane-1-carboxylic acid deaminase production	*Brassica napus*	NM	[172]
*K*. *ascorbata* SUD165, SUD165/26	Increased biomass; Aminocyclopropane-1-carboxylic acid deaminase, siderophores production	*Brassica juncea*	NM	[172]
*Enterobacter cloacae* CAL2	Increased biomass; Aminocyclopropane-1-carboxylic acid deaminase production	*Brassica napus*	Enterobacter cloacae CAL2	[172]
*B*. *subtilis* SJ-101	Indole acetic acid production; phosphate solubilization	*Brassica juncea*	NM	[172]
*Enterobacter* sp. NBRI K28	Increased biomass; indole acetic acid, siderophores, Aminocyclopropane-1-carboxylic acid deaminase production; phosphate solubilization	*Brassica juncea*	NM	[172]
*Bacillus* sp. J119	Increased biomass, indole acetic acid, siderophores production; biosurfactant production	*Brassica napus*	NM	[172]
*P*. *aeruginosa* MKRh3	Increased biomass and rooting, indole acetic acid, siderophores, aminocyclopropane-1-carboxylic acid deaminase production; phosphate solubilization	*Vigna mungo*	NM	[172]
*Bacillus* sp. J119	I indole acetic acid, siderophores production; biosurfactant production	*Brassica napus*	NM	[172]
*Pseudomonas* sp. M6, *Pseudomonas jessenii* M15	Increased biomass; indole acetic acid, aminocyclopropane-1-carboxylic acid deaminase production; phosphate solubilization	*Ricinus communis*	NM	[172]
*Achromobacter xylosoxidans* Ax10	Increased root and shoot length and biomass; aminocyclopropane-1-carboxylic acid deaminase production; phosphate solubilization, indole acetic acid production	*Brassica juncea*	NM	[172]
*Enterobacter aerogenes*, *Rahnella aquatilis*	Increased biomass and metal uptake; indole acetic acid, siderophores, aminocyclopropane-1-carboxylic acid deaminase production, phosphate solubilization	*Brassica juncea*	NM	[172]
*B*. *subtilis* SJ-101	indole acetic acid, P solubilization	*Brassica juncea*	NM	[172]
*P*. *putida* ARB86	Increased biomass and chlorophyll content	*Arabidopsis thaliana*	NM	[172]
*Bradyrhizobium* sp. RM8	Increased growth, seed yield, seed protein, nodule number, plant nutrition, indole acetic acid, siderophores, hydrogen cyanide, ammonia production	*Vigna mungo*	NM	[172]
*Rhizobium* sp. RP5	Increased biomass, nodule number and plant nutrition, indole acetic acid, siderophores production	*Pisum sativum*	NM	[172]
*Enterobacter* sp. NBRI K28	Increased biomass, protein, and chlorophyll content, indole acetic acid, siderophores, Aminocyclopropane-1-carboxylic acid deaminase production; P solubilization	*Brassica juncea*	NM	[172]
*Pseudomonas* sp. 29C, *Bacillus* sp. 4C	Increased biomass; indole acetic acid, siderophores production, Aminocyclopropane-1-carboxylic acid deaminase, P solubilization	*Brassica juncea*	NM	[172]
*Pseudomonas* sp. M6, *Pseudomonas jessenii* M15	Increased biomass; indole acetic acid, aminocyclopropane-1-carboxylic acid deaminase production; P solubilization	*Ricinus communis*	NM	[172]
*Pseudomonas* sp.	Increased biomass; siderophores production	*Cicer arietinum*	NM	[172]
*Enterobacter erogenes*, *Rahnella aquatilis*	Increased biomass, indole acetic acid, siderophores, aminocyclopropane-1-carboxylic acid deaminase production, P solubilization	*Brassica juncea*	NM	[172]
*Psychrobacter* sp. SRA1 and SRA2, *Bacillus cereus* SRA10	Increased biomass, indole acetic acid, siderophores, aminocyclopropane-1-carboxylic acid deaminase production. P solubilization	*Brassica juncea*, *B*. *oxyrrhina*	NM	[172]
*Rahnella* sp. JN6	Indole acetic acid, aminocyclopropane-1-carboxylic acid deaminase, siderophores, phosphate solubilization	*B*. *napus*	Root, shoot	[172]
*Psuedomonas aspleni*	Increased biomass; indole acetic acid production	*Brassica napus*	NM	[172]
*Variovorax paradoxus*, *Rhodoccus* sp., *Flavobacterium* sp.	Increased root length; indole acetic acid, siderophores, production Aminocyclopropane-1-carboxylic acid deaminase production	*Brassica juncea*	NM	[172]
*Pseudomonas fluorescens*, *P*. *putida*	Increased seed germination and growth	*Brassica napus*	NM	[172]
*P*. *putida* UW4, *P*. *putida* HS-2	Increased biomass in the field; indole acetic acid, Aminocyclopropane-1-carboxylic acid deaminase production	*Brassica napus*	NM	[172]
*Sinorhizobium* sp. Pb002	Aminocyclopropane-1-carboxylic acid deaminase production	*Brassica juncea*	NM	[172]
*Azotobacter chroococcum* HKN-5, *B*. *megaterium* HKP-1, *B*. *mucilaginosus* HKK-1	Increased biomass	*Brassica juncea*	NM	[172]
*Pseudomonas* sp. RJ10, *Bacillus* sp. RJ16	Increased biomass; indole acetic acid production	*Brassica napus*	NM	[172]
*Mesorhizobium huakuii* subsp. *rengei* B3	Phytochelatin and metallothionein production	*Astragalus sinicus*	NM	[172]
*Burkholderia cepacia*	Increased biomass	*Sedum alfredii*	NM	[172]
*P*. *putida ARB86*	Increased biomass and chlorophyll content	*Arabidopsis thaliana*	NM	[172]
*P*. *putida* HS-2	Increased seed germination and biomass; siderophores, indole acetic acid, aminocyclopropane-1-carboxylic acid deaminase production	*Brassica napus*	NM	[172]
*Pseudomonas* sp. 29C, *Bacillus* sp. 4C	Increased biomass; indole acetic acid, siderophores, aminocyclopropane-1-carboxylic acid deaminase production; phosphate solubilization	*Brassica juncea*	NM	[172]
*Proteus vulgaris* KNP3	Increased germination, biomass, and chlorophyll	*Cajanus cajan*	NM	[172]
*Pseudomonas* sp.	Increased biomass siderophores production	*Cicer arietinum*	NM	[172]
*P*. *fluorescens* G10, *Microbacterium* sp. G16	Increased biomass; indole acetic acid, siderophores, aminocyclopropane-1-carboxylic acid deaminase production	*Brassica napus*	NM	[172]
*Bacillus edaphicus* NBT	Increased biomass; indole acetic acid, siderophores, aminocyclopropane-1-carboxylic acid deaminase production	*Brassica juncea*	NM	[172]
*Flavobacterium* sp.	Increased root length, biomass	*Orychophragmus violaceus*	NM	[172]
*Kluyvera ascorbata* SSUD165	Increased biomass; aminocyclopropane-1-carboxylic acid deaminase production	*Brassica napus*	NM	[172]
*K*. *ascorbata* SUD165, SUD165/26	Increased biomass; aminocyclopropane-1-carboxylic acid deaminase, siderophores production	*Brassica napus*	NM	[172]
*P*. *fluorescens*, *P*. *putida*	Increased seed germination and growth	*Brassica napus*	NM	[172]
*P*. *putida* HS-2	Increased seed germination and biomass; siderophores, indole acetic acid, aminocyclopropane-1-carboxylic acid deaminase production	*Brassica napus*	NM	[172]
*Different endophytic bacteria*	Na+/K+ accumulation	*Salicornia*, *Arthrocnemum*, *Haloxylon*, *Sesuvium*, *Suaeda*, *Aeluropus*, *Heleochloa*, *Atriplex*, *and Salvadoraet*	shoot and root	[173]
*Pseudoalteromonas maricaloris*	1-aminocyclopropane-1-carboxylic acid Deaminase production	*Avicennia marina*	Rhizosphere	[174]

NM = not mentioned.

**Table 3:** Phytoremediation bacteria and associated secondary metabolites from medicinal and herbal plant species.

Isolated Bacterial Species	Biochemical/Physiological Activity	Host Medicinal Plant Species	Isolated Plant Part/Region	References
*Pseudomonas aeruginosa*, *Pseudomonas savastanoi*	Mono- and dechlorinated, benzoic acids production	*Elymus dauricus*		[211]
*Methylobacterium populi*	Methane; 2,4,6-trinitrotoluene; hexahydro-1,3,5-trinitro-1,3,5-triazene; octahydro-1,3,5,7-tetranitro-1,3,5-tetrazocine; benzene, toluene, ethylbenzene, xylene production	*Populus deltoids x nigra*	NM	[212]
*Pseudomonas* sp., *Bacillus cepacia*	Benzene, toluene, ethylbenzene, xylene production	*Populus* sp.	NM	[213]
*Burkholderia cepacia*	Volatile organic compounds, toluene production	*Lupinus luteus*	NM	[214]
*Pseudomonas putida*	2,4-Dichlorophenoxyacetic acid production	*Populus* sp., *Trichocarpa deltoides* cv. *Hoogvorst*	Stem	[213]
*Proteobacteria* sp.	NM	*Populus nigra* cv. *Brandaris*	Root, shoot	[215]
*Proteobacteria* sp.	NM	*Lolium multiflorum* cv. *Lolita*	Root, shoot	[215]
*Proteobacteria* sp.	NM	*Medicago sativa* cv. Europ	Root, shoot	[215]
*Pseudomonas! uorescens* G10, *Microbacterium* sp. G16 (EN)	indole acetic acid, siderophores, and 1-aminocyclopropane-1-carboxylate deaminase production	*Brassica napus*	Root	[216]
*Arthrobacter* sp. MT16, *Microbacterium* sp. JYC17, *Pseudomonas chlororaphis* SZY6	NM	*Brassica napus*	Whole plant	[217]
*Firmicutes* sp., *Actinobacteria* sp., *Proteobacteria* sp. (EN)	NM	*Elsholtzia splendens*, *Commelina communis*	Whole plant	[218]
*Enterobacter* sp.	NM	*Allium macrostemon*	Stem	[219]
*Sphingopyxis* sp., *Pseudomonas* sp.	NM	*Lolium multiflorum*, *Lotus corniculatus*	Root, shoot	[220]
*Achromobacter xylosoxidans*	NM	*Phragmites australis*, *Ipomoea* *aquatica*, *Vetiveria zizanioides*	Root	[221]
*Burkholderia macroides*	NM	*Populus* cv. *Hoogvorst*	Stem	[222]
*Pseudomonas putida*	NM	*Populus trichocarpa × deltoides* cv. Hoogvorst	Stem	[222]
*Pseudomonas tolaasii*, *P*. *jessenii*, *Ps*. *Rhodesiae*, *P*. *plecoglossicida*, *P*. *veronii*, *P*. *fulva*, *P*. *oryzihabitans*, *Acinetobacter lwoffi*, *A*. *nicotianae*, *Bacillus megaterium*, *Paenibacillus amylolyticus*	NM	*Populus* sp. cv. *Hazendans*	Root, stem	[15]
*Pseudomonas putida*	NM	*Populus* sp.	NM	[223]
*Staphylococcus* sp., *Microbacterium* sp., *Pseudomonas* sp., *Staphylococcus* sp., *Curtobacterium* sp., *Microbacterium* sp., *Curtobacterium* sp., *Staphylococcus* sp., *Bacillus* sp., *Arthrobacter* sp., *Pseudomonas* sp., *Curtobacterium* sp., *Microbacterium* sp., *Paenibacillus* sp., *Leifsonia* sp.	NM	*Alyssum bertolonii*	Leaf, stem, root	[224]
*Bacillus* sp.	NM	*Alnus firma*	Root	[225]
*Sphingomonas* sp., *Methylobacterium* sp., *Sphingobacterium multivorum*, *Phyllobacterium* sp., *Devosia* sp., *Afibia* sp., *Sphingomonas* sp., *Rhodococcus* sp.	NM	*Thlaspi caerulescens*	Stem, root	[226]
*Alphaproteobacteria* sp., *Holophaga* sp., *Acidobacterium* sp., *Betaproteobacteria* sp., *Gammaproteobacteria* sp., *Bacillus* sp., *Blastococcus* sp., *Propionibacterium acnes*, *Flavobacterium* sp., *Desulfitobacterium metallireductans*, *M*. *mesophilicum*, *M*. *extorquens*, *Sphingomonas* sp., *Curtobacterium* sp., *Plantibacter flavus*, *Rhodococcus* sp.	NM	*Thlaspi goesingense*	Stem	[227]
*Microbacterium* sp., *Bacillus* sp., *Arthrobacter* sp., *Flavobacterium* sp., *Chryseobacterium* sp., *Agrobacterium* sp., *Sphingomonas* sp., *Pseudomonas* sp., *Serratia* sp., *Curtobacteriu* sp.	NM	*Solanum nigrum*	Root, stem, leaf	[228]
*Acinetobacter* sp., *Bacillus* sp.	Aminocyclopropane-1-carboxylic acid deaminase production	*Commelina communis*	Root, stem, leaf	[229]
*Acinetobacter* sp., *Moraxella* sp., *Serratias* sp., *Herbaspirillum* sp., *Bukholderia* sp., *Paracoccus* sp., *Sphingomonas* sp., *Exiguobacterium* sp., *Bacillus* sp., *Arthrobacter* sp., *Microbacterium* sp., *Micrococcus* sp.	NM	*Elsholtzia splendens* and *Commelina communis*	Root, stem, leaf	[230]
*Enterobacter* sp.	NM	*Allium macrostemon*	Stem	[231]
*Microbacterium* sp., *Frigoribacterium* sp., *Methylobacterium* sp., *Sphingomonas* sp.	NM	*Salix caprea*	Stem, leaf	[232]
*Pseudomonas frederisksbergensis* sp. *Veronii*, *P*. *Putida*, *Arthrobacter illicis*, *A*. *histidinolovorans*	NM	*Populus* sp. cv. *Hazendans* and *Populus* sp. cv. Hoogvorst	Root, stem	[168]
*Staphylococcus* sp., *Microbacterium* sp., *Pseudomonas* sp., *Curtobacterium* sp., *Bacillus* sp., *Arthrobacter* sp., *Paenibacillus* sp., *Leifsonia* sp.	NM	*A*. *bertolonii*	Whole plant	[168]
*Pseudomonas* sp., *Streptomyces* sp.	NM	*A*. *bertolonii*	Rhizosphere	[168]
*Holophaga* sp., *Acidobacterium* sp., *Gammaproteobacteria* sp., *Betaproteobacteria* sp., *Alphaproteobacteria* sp., *Verrucomicrobia* sp., *Gemmatimonadetes* sp.	NM	*Thlaspi goesingense*	Rhizosphere, shoot	[168]
*Pseudomonas fluorescens* G10, *Microbacterium* sp. G16	Indole acetic acid, aminocyclopro-pane-1-carboxylic acid deaminase, siderophore production	*Brassica napus*	Root, shoot	[172]
*Enterobactor cloacae* CAL2	Indole acetic acid, aminocyclopropane-1-carboxylic acid deaminase, siderophores, antibiotics production	*B*. *napus*	Root, shoot	[172]
*Cupriavidus taiwanensis*	Biodegradation, biosorption, release of extracellular products	*Mimosa pudica*	Root, shoot	[172]
*Bacillus thuringiensis* GDB-1	Aminocyclopropane-1-carboxylic acid deaminase, indole acetic acid, Siderophores	*Alnus firma*	Root, shoot	[172]
*B*. *pumilus* E2S2, *Bacillus* sp. *E1S2*	Indole acetic acid, aminocyclo-pro-pane-1-carboxylic acid deaminase, siderophores	*Sedum plumbizincicola*	Root, shoot	[172]
*Pseudomonas tolaasii* ACC23, *P*. *fluorescens* ACC9	Aminocyclo-pro-pane-1-carboxylic acid deaminase, siderophores, and indole acetic acid production	*B*. *napus*	Root, shoot	[172]
*Pseudomonas* sp. LK9	Biosurfactants, siderophores, organic acids production	*Solanum nigrum*	Root, stem, leaf	[172]
*Pseudomonas* sp. PsM6, *P*. *Jessenii* PjM15	Biosorption, mobilization, aminocyclopropane-1-carboxylic acid deaminase, indole acetic acid, siderophores production	*Ricinus communis*	Root, stem, leaf	[172]
*Rahnella* sp. JN6	Indole acetic acid, aminocyclopropane-1-carboxylic acid deaminase, siderophores	*B*. *napus*	Root, shoot	[172]
*Bacilus subtilis*, *B*. *cereus*, *Flavobacterium* sp., *Pseudomonas aeroginosa*	Aminocyclopropane-1-carboxylic acid deaminase, indole acetic acid, siderophores	*Orycoprhagmus* *violaceus*	Root, shoot	[172]
*Pseudomonas veronii*	indole acetic acid production, decrease soil pH, supply P, and Fe	*S*. *alfredii*	Root, shoot	[172]
*Staphylococcus arlettae* NBRIEAG-6	indole acetic acid, siderophores, Aminocyclopropane-1-carboxylic acid deaminase production	*Brassica juncea*	Root, shoot	[172]
*Achromobacter xylosoxidans* Ax10	Aminocyclopropane-1-carboxylic acid deaminase, indole acetic acid production	*B*. *Juncea*	Root, shoot	[172]
*Psychrobacter* sp. SRA1, *Bacillus cereus* SRA10	Aminocyclopropane-1-carboxylic acid deaminase, indole acetic acid, Ni mobilization	*B*. *Juncea*	Root, shoot	[172]
*Pseudomonas* sp. SRI2, *Psychrobacter* sp. SRS8, *Bacillus* sp. SN9	Aminocyclopropane-1-carboxylic acid deaminase, indole acetic acid production; Ni mobilization	*B*. *Juncea*	Root, shoot	[172]
*Pseudomonas* sp. SRI2, *Psychrobacter* sp. SRS8, *Bacillus* sp. SN9	Aminocyclopropane-1-carboxylic acid deaminase, indole acetic acid production; Ni mobilization	*Brassica oxyrrhina*	Root, shoot	[172]
*Paenibacillus macerans* NBRFT5, *Bacillus endophyticus* NBRFT4, *B*. *pumilus* NBRFT9	Siderophores, organic acids, protons, and other non-specified enzymes production	*B*. *Juncea*	Root, stem, leaf	[172]
*Rhizobium leguminozarum*	Metal chelation	*B*. *Juncea*	Whole plant	[172]
*Azotobacter chroococcum* HKN-5, *Bacillus megaterium* HKP-1, *Bacillus mucilaginosun* HKK-1	indole acetic acid, gibberellins production	*B*. *Juncea*	Shoot	[172]
*Pantoea agglomerans* Jp3-3, *Pseudomonas thivervalensis* Y1-3-9	Indole acetic acid, siderophores, Aminocyclopropane-1-carboxylic acid deaminase production	*B*. *napus*	Root, shoot	[172]
*Enterobacter* sp. JYX7, *Klebsiella* sp. JYX10	Indole acetic acid, siderophores, Aminocyclopropane-1-carboxylic acid deaminase production	*Polygonum pubescens*	Root, shoot	[172]
*Rahnella* sp.	Indole acetic acid, siderophores, aminocyclopropane-1-carboxylic acid deaminase production	*Amaranthus hypochondriacus*, *A*. *mangostanus*, *Solanum nigrum*	Root, shoot	[172]
*Kluyvera* sp.	Aminocyclopropane-1-carboxylic acid deaminase production	*Brassica napus*	NM	[172]
*Kluyver spp*.	Aminocyclopropane-1-carboxylic acid deaminase, siderophores production	*Brassica juncea*	NM	[172]
*Brevibacillus* sp.	Decreased lead uptake; indole acetic acid production	*Trifolium pratense*	NM	[172]
*Enterobacter cloacae* CAL2	Aminocyclopropane-1-carboxylic acid deaminase production	*Brassica napus*	NM	[172]
*Microbacterium arabinogalactanolyticum*	Increased nickel uptake	*Alyssum murale*	NM	[172]
*Psuedomonas* sp.	Increased biomass; indole acetic acid production	*Brassica napus*	NM	[172]
*Variovorax paradoxus*, *Rhodoccus* sp., *Flavobacterium* sp.	Increased root length; Indole acetic acid, siderophores, production Aminocyclopropane-1-carboxylic acid deaminase production	*Brassica juncea*	NM	[172]
*Pseudomonas fluorescens*, *P*. *putida*	Increased seed germination and growth	*Brassica napus*	NM	[172]
*P*. *putida* UW4, *P*. *putida* HS-2	Increased biomass in the field; indole acetic acid, Aminocyclopropane-1-carboxylic acid deaminase production	*Brassica napus*	NM	[172]
*Sinorhizobium* sp. Pb002	Increased plant survival and lead uptake; aminocyclopropane-1-carboxylic acid deaminase production	*Brassica juncea*	NM	[172]
*Azotobacter chroococcum* HKN-5, *B*. *megaterium* HKP-1, *B*. *mucilaginosus* HKK-1	Increased biomass and metal bioavailability	*Brassica juncea*	NM	[172]
*B*. *subtilis* SJ-101	Increased nickel uptake; indole acetic acid production	*Brassica juncea*	NM	[172]
*Pseudomonas* sp. RJ10, *Bacillus* sp. RJ16	Increased biomass and metal uptake; indole acetic acid production	*Brassica napus*	NM	[172]
*Mesorhizobium huakuii* subsp. *Rengei* B3	Increased metal accumulation: bacterium expresses phytochelatin, and metallothionein	*Astragalus sinicus*	NM	[172]
*Burkholderia cepacia*	Increased biomass, metal uptake, and translocation of metal to shoots	*Sedum alfredii*	NM	[172]
*P*. *putida ARB86*	Increased biomass and chlorophyll content	*Arabidopsis thaliana*	NM	[172]
*Enterobacter* sp. NBRI K28	Increased metal uptake; indole acetic acid, siderophores, Aminocyclopropane-1-carboxylic acid deaminase production	*Brassica juncea*	NM	[172]
*P*. *putida* HS-2	Increased seed germination and biomass; siderophores, indole acetic acid, Aminocyclopropane-1-carboxylic acid deaminase production	*Brassica napus*	NM	[172]
*Bacillus* sp. J119	Increased cadmium uptake, indole acetic acid, siderophores production; biosurfactant production	*Brassica napus*	NM	[172]
*P*. *aeruginosa* MKRh3	Decreased cadmium uptake; indole acetic acid, siderophores, Aminocyclopropane-1-carboxylic acid deaminase production	*Vigna mungo*	NM	[172]
*Pseudomonas* sp. 29C, *Bacillus* sp. 4C	Increased biomass; indole acetic acid, siderophores, aminocyclopropane-1-carboxylic acid deaminase production;	*Brassica juncea*	NM	[172]
*Pseudomonas* sp. M6, *Pseudomonas jessenii* M15	Increased biomass; indole acetic acid, Aminocyclopropane-1-carboxylic acid deaminase production	*Ricinus communis*	NM	[172]
*Proteus vulgaris* KNP3	Increased germination, biomass, and chlorophyll, and decreased metal uptake	*Cajanus cajan*	NM	[172]
*Pseudomonas* sp.	Increased biomass and decreased metal uptake; siderophores production	*Cicer arietinum*	NM	[172]
*P*. *fluorescens* G10, *Microbacterium* sp. G16	Increased biomass and metal uptake; indole acetic acid, siderophores, aminocyclopropane-1-carboxylic acid deaminase production	*Brassica napus*	NM	[172]
*Bacillus edaphicus* NBT	Increased biomass; indole acetic acid, siderophores, aminocyclopropane-1-carboxylic acid deaminase production	*Brassica juncea*	NM	[172]
*Flavobacterium* sp.	Increased root length, biomass, metal uptake	*Orychophragmus violaceus*	NM	[172]
*Achromobacter xylosoxidans* Ax10	Increased root and shoot length and biomass; aminocyclopropane-1-carboxylic acid deaminase production; indole acetic acid production	*Brassica juncea*	NM	[172]
*Enterobacter aerogenes*, *Rahnella aquatilis*	Increased biomass and metal uptake; indole acetic acid, siderophores, aminocyclopropane-1-carboxylic acid deaminase production	*Brassica juncea*	NM	[172]
*Kluyvera* sp.	Increased biomass; aminocyclopropane-1-carboxylic acid deaminase production	*Brassica napus*	NM	[172]
*Kluyvera* sp.	Increased biomass; aminocyclopropane-1-carboxylic acid deaminase, siderophores production	*Brassica napus*	NM	[172]
*Microbacterium* *arabinogalactanolyticum*	Increased Ni uptake	*Alyssum murale*	NM	[172]
*P*. *fluorescens*, *P*. *putida*	Increased seed germination and growth	*Brassica napus*	NM	[172]
*P*. *putida* UW4, *P*. *putida* HS-2	Indole acetic acid, aminocyclopropane-1-carboxylic acid deaminase production	*Brassica napus*	NM	[172]
*B*. *subtilis* SJ-101	Increased Ni, indole acetic acid production	*Brassica juncea*	NM	[172]
*Bradyrhizobium* sp. RM8	Decreased Ni toxicity and uptake; indole acetic acid, siderophores, hydrogen cyanide, ammonia production	*Vigna mungo*	NM	[172]
*Rhizobium* sp. RP5	Decreased Ni, Zn uptake, and toxicity; indole acetic acid, siderophores production	*Pisum sativum*	NM	[172]
*Enterobacter* sp. NBRI K28	Increased Ni, Zn, Cr uptake; I indole acetic acid, siderophores, Aminocyclopropane-1-carboxylic acid deaminase production	*Brassica juncea*	NM	[172]
*P*. *putida* HS-2	Siderophores, indole acetic acid, Aminocyclopropane-1-carboxylic acid deaminase production	*Brassica napus*	NM	[172]
*Pseudomonas* sp. 29C, *Bacillus* sp. 4C	indole acetic acid, siderophores production, Aminocyclopropane-1-carboxylic acid deaminase	*Brassica juncea*	NM	[172]
*Pseudomonas* sp. M6, *Pseudomonas jessenii* M15	indole acetic acid, aminocyclopropane-1-carboxylic acid deaminase production	*Ricinus communis*	NM	[172]
*Pseudomonas* sp.	Decreased Ni uptake; siderophores production	*Cicer arietinum*	NM	[172]
*Enterobacter erogenes*, *Rahnella aquatilis*	Increased Ni, Cr uptake; indole acetic acid, siderophores, aminocyclopropane-1-carboxylic acid deaminase production	*Brassica juncea*	NM	[172]
*Psychrobacter* sp. SRA1 and SRA2, *Bacillus cereus* SRA10	Increased Ni bioavailability and uptake; indole acetic acid, siderophores, aminocyclopropane-1-carboxylic acid deaminase production	*Brassica juncea*, *B*. *oxyrrhina*	NM	[172]
*Brachybacterium*	Production of halotolerant properties	*Salicornia brachiate r*	Rhizosphere	[233]
*Pseudomonas* sp. SRI2, *Psychrobacter* sp. SRS8 and *Bacillus* sp. SN9	NM	*Brassica juncea*, *B*. *oxyrrhina*	NM	[172]

NM = not mentioned.

## Data Availability

Data is unavailable.
